# Neuroligin 1 Regulates Autistic‐Like Repetitive Behavior through Modulating the Activity of Striatal D2 Receptor‐Expressing Medium Spiny Neurons

**DOI:** 10.1002/advs.202410728

**Published:** 2024-12-11

**Authors:** Dandan Lv, An Liu, Ziyue Yi, Mingdao Mu, Miao Wu, Xingcan Li, Kun Cao, Ruining Liu, Zhengping Jia, Junhai Han, Wei Xie

**Affiliations:** ^1^ The Key Laboratory of Developmental Genes and Human Disease The School of Life Science and Technology Southeast University 2 Sipailou Road Nanjing 210096 China; ^2^ Institute for Brain and Intelligence Southeast University 2 Sipailou Road Nanjing 210096 China; ^3^ Shenzhen Research Institute Southeast University 19 Gaoxin South 4th Road Shenzhen 518063 China; ^4^ School of Medicine Southeast University 87 Dingjiaqiao Road Nanjing 210009 China; ^5^ Neurosciences & Mental Health The Hospital for Sick Children 555 University Ave. Toronto Ontario M5G 1×8 Canada; ^6^ Department of Physiology Faculty of Medicine University of Toronto 1 King's College Circle Toronto Ontario M5S 1A8 Canada; ^7^ Jiangsu Co‐innovation Center of Neuroregeneration Southeast University 2 Sipailou Road Nanjing 210096 China

**Keywords:** Autism Spectrum Disorder (ASD), D2‐MSN, neuroligin1, restricted and repetitive behavior (RRB), striatum

## Abstract

Restricted and repetitive behavior (RRB) is a primary symptom of autism spectrum disorder (ASD), which poses a significant risk to individuals' health and is becoming increasingly prevalent. However, the specific cellular and neural circuit mechanisms underlying the generation of RRB remain unclear. In this study, it is reported that the absence of the ASD‐related protein Neuroligin 1 (NLGN1) in dopamine receptor D2‐expressing medium spiny neurons (D2‐MSNs) in the dorsal striatum is associated with the duration and frequency of self‐grooming and digging behaviors. The *Nlgn1*‐deficient D2‐MSNs are hyperactivated, which correlates with excessive self‐grooming and digging behaviors. Inhibiting the activity of D2‐MSNs reduces the duration and frequency of these RRBs. Furthermore, it is demonstrated that the generation of self‐grooming and digging behaviors depends on distinct patterns of D2‐MSN activity. Finally, through single‐nucleus RNA sequencing (sn‐RNAseq) and protein detection verification, it is revealed that the overactivation of protein kinase C (PKC) in *Nlgn1*‐deficient mice contributes to excessive repetitive behaviors and increased neuronal excitability. In this study, potential mechanisms are proposed for the generation of self‐grooming and digging behaviors, as well as suggest possible treatments and interventions ASD.

## Graphical abstract

The present study reports that ASD‐associated protein NLGN1 in WT dorsal striatum D2‐MSN inhibits PKC activity and regulates neuronal excitability; in *Nlgn1* KO mice, PKC activity in D2‐MSNs increases, which enhances neuronal excitability and leads to RRBs. They hope this study will aid in the treatment and intervention of ASD.

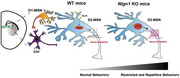

## 1 Introduction

Autism spectrum disorders (ASD) are a group of heterogeneous neurodevelopmental disorders characterized by persistent deficits in social communication and social interaction, as well as restricted and repetitive behaviors (RRBs), interests, or activities.^[^ ^1^, ^2^ ^]^ The RRBs refer to various rigid behavioral patterns, including stereotypes, self‐injury, insistence on sameness, rituals, and compulsive behaviors. Given the increasing incidence rate and health hazards of ASD, the research on the cellular and molecular mechanisms that cause ASD, particularly those driving RRBs has been the focus of attention.^[^ ^3^, ^4^ ^]^

The striatum, as the input nucleus of the basal ganglia (BG), receives glutamate inputs from cortical, thalamic, and other brain regions, as well as dopaminergic inputs from substantia nigra pars compacta (SNc) and ventral tegmental area (VTA),^[^ ^5^, ^6^ ^]^ and participates in autonomous movement controlling, motor planning, action selection, reward‐guided learning, as well as RRBs.^[^ ^7^, ^8^ ^]^ Dopamine receptor 1 (D1) or dopamine receptor 2 (D2) expressed medium spiny neurons (D1‐MSNs or D2‐MSNs) make up ≈95% of the striatal neurons. The remaining portion is composed of cholinergic and aspiny GABAergic interneurons, which are responsible for strongly regulating the input and output of the MSNs.^[^ ^5^, ^9^ ^]^ After integrating information input, D1‐MSN and D2‐MSN project to downstream brain regions through the so‐called direct or indirect pathways, targeting globus pallidus pars interna/substantia nigra pars reticulata (GPi/SNr) and globus pallidus pars externa (GPe) respectively.^[^ ^5^, ^7^ ^]^ Previous rodent investigations have demonstrated that certain repeated behaviors, such as self‐grooming, can activate striatal neurons and be disrupted by striatal injury.^[^ ^10^, ^11^ ^]^ The deletion or mutation of ASD‐related genes, such as *Shanks*, *Fmr1*, and *Cntnaps* in MSN are closely related to excessive RRBs, indicating the critical roles of the striatum in RRB controlling.^[^ ^12^, ^13^, ^14^ ^]^ However, the specific mechanisms by which MSNs integrate information input and guide distinct behaviors are still less clear.

The Neuroligins (NLGNs) family is a class of postsynaptic adhesion molecules strongly associated with ASD,^[^ ^15^, ^16^, ^17^, ^18^ ^]^ among which NLGN1 is reported restricted to the excitatory synapse, and well known for its roles in glutamatergic synaptic maturation and synaptic transmission in pyramidal neurons.^[^ ^19^, ^20^, ^21^, ^22^ ^]^ However, little is known about its function in other types of cell. We and others previously reported excessive self‐grooming behavior in *Nlgn1* homozygous knockout (KO) and heterozygous (Het) mice, which is a kind of widely studied RRB.^[^ ^23^, ^24^, ^25^ ^]^ Nevertheless, the mechanism by which NLGN1 loss induces RRBs remains unclear.

In this study, we report that the deletion of NLGN1 in dorsal striatal D2‐MSN increases the likelihood of self‐grooming and digging behaviors, thereby inducing excessive RRBs. The two distinct repetitive behaviors were driven by the overactivation of D2‐MSN under different activity patterns. Through single‐nucleus RNA sequencing (snRNA‐seq) and protein assays, we further demonstrated that the excessive neural excitation and the occurrence of RRBs were associated with the overactivation of protein kinase C (PKC) in D2‐MSN.

## 2 Results

### 2.1 Suppression of NLGN1 in Striatum D2‐MSN Promotes RRBs

**Figure 1:**
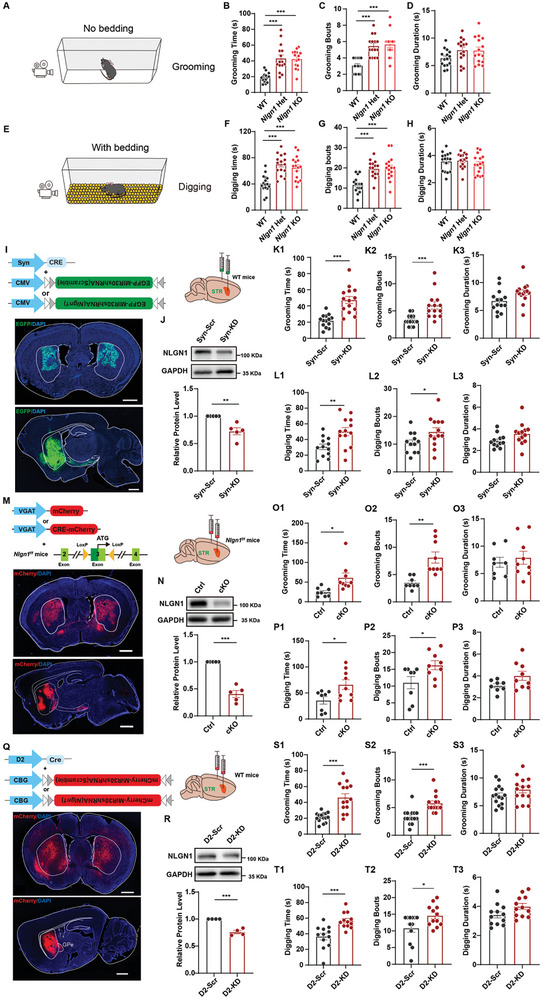
Knocking down *Nlgn1* in D2‐MSN promotes RRBs. A) Illustration of self‐grooming behavior recording. B‐D) Statistical graphs of self‐grooming time (B), bouts (C), and average grooming duration per bout (D) in WT, *Nlgn1* heterozygous (*Nlgn1* Het) and homozygous (*Nlgn1* KO) mice. E) Illustration of spontaneous digging behavior recording. F‐H) Statistical graphs of digging time (F), bouts (G), and average digging duration per bout (H) in WT, *Nlgn1* Het and *Nlgn1* KO mice. I) Illustration and sample images of Syn‐driven *Nlgn1* KD (knockdown) virus expression. Scale bar: 1mm. J) Representative images (upper) and statistical graph (lower) of NLGN1 protein expression in striatum tissues from Syn‐driven scramble (Scr) and *Nlgn1* KD viruses‐expressed mice. K1‐K3) Statistical graphs of self‐grooming time (K1), bouts (K2), and average grooming duration per bout (K3) in mice depicted in panel (J). L1‐L3) Statistical graphs of digging time (L1), bouts (L2), and average digging duration per bout (L3) in mice depicted in panel (J). M) Illustration of construction strategy of *Nlgn1^f/f^* mice and sample images of VGAT‐mCherry (control, Ctrl) or VGAT‐CRE‐mCherry (conditional knockout, cKO) virus expressed in the striatum of *Nlgn1^f/f^* mice. Scale bar: 1mm. N) Representative images (upper) and statistical graph (lower) of NLGN1 protein in striatal tissues from *Nlgn1^f/f^* mice depicted in panel (M). O1‐O3) Statistical graphs of self‐grooming time (O1), bouts (O2), and average grooming duration per bout (O3) in *Nlgn1^f/f^* mice depicted in panel (M). P1‐P3) Statistical graphs of digging time (P1), bouts (P2), and average digging duration per bout (P3) in *Nlgn1^f/f^* mice depicted in panel (M). Q) Illustration and sample images of D2‐driven *Nlgn1* KD viruses expressed mice. Scale bar: 1mm. R) Representative images (upper) and statistical graph (lower) of NLGN1 protein expression in striatal tissues from mice depicted in panel (Q). S1‐S3) Statistical graphs of self‐grooming time (S1), bouts (S2), and average grooming duration per bout (S3) in mice depicted in panel (Q). T1‐T3) Statistical graphs of digging time (T1), bouts (T2), and average digging duration per bout (T3) in mice depicted in panel (Q). Data represent mean ± SEM; Two‐way ANOVA with Fisher's LSD post hoc test for panel C‐H, two‐tailed unpaired *t*‐test for panel J‐L, N‐ P and R‐T; For all the panels, dots represent individual mice. *p < 0.05; **p < 0.01; ***p < 0.001. Also see Figures S1,S2 and Tables S1,S2 (Supporting Information).

Excessive RRB is common in both patients with autism spectrum disorder (ASD) and animal models. Our previous research demonstrated that both *Nlgn1* KO and Het mice exhibit significant self‐grooming behavior (**Figure** **1**A–C).^[^ ^24^ ^]^ However, the specific brain regions and cellular mechanisms involved remain unclear. Additionally, we discovered that *Nlgn1*‐deficient mice displayed excessive digging behavior when introduced to bedded cages (Figure 1E–H). Our results indicated a significant increase in the frequency of self‐grooming and digging sessions, while the duration of each session remained normal (Figure 1B–D and F–H). Further analysis revealed that these changes were consistently observed in both male and female *Nlgn1*‐deficient mice (Figure S1A–D, Supporting Information). These findings suggest that *Nlgn1* deficiency increases the likelihood of developing repetitive behaviors, resulting in an overall increase in total RRB time.

Previous studies have reported that brain regions such as the central amygdala (CeA), paraventricular hypothalamic nucleus (PVH), medial prefrontal cortex (mPFC), and orbitofrontal cortex (OFC) are involved in regulating the generation or pattern of RRBs.^[^ ^8^, ^10^ ^]^ To determine if the lack of NLGN1 in these brain regions results in increased RRB, we administered AAV viruses with Synapsin1 (Syn) promoter‐driven Cre (rAAV‐hSyn‐CRE) and Cre‐dependent *Nlgn1*‐shRNA (rAAV‐CMV‐bGlobin‐Flex‐EGFP‐MIR30shRNA(*Nlgn1*)) to achieve knockdown (KD) NLGN1 in neurons within these brain regions (Figure S1E, Supporting Information). We conducted behavioral tests five weeks later, and the results indicated that inhibiting *Nlgn1* specifically in striatal neurons led to excessive self‐grooming and digging behaviors (Figure 1I‐K1, L1; Figure S1F–G, Supporting Information). We further analyzed the reasons for the increased RRB in mice with NLGN1 suppression in striatal neurons. The results showed that, similar to *Nlgn1* KO mice, there was a significant increase in the frequency of self‐grooming and digging behaviors, while the duration of each behavior remained unchanged. (Figure 1K2‐K3 and L2‐L3).

Given that the primary cells in the striatum are GABAergic MSN neurons, we investigated whether the excessive RRBs were caused by a deficiency of *Nlgn1* in these GABAergic neurons. To do this, we bilaterally injected a GABAergic‐specific vGAT‐driven Cre (rAAV‐VGAT1‐CRE‐mCherry) virus into the striatal region of floxed *Nlgn1* (*Nlgn1^f/f^*) mice (Figure 1M,N). Five weeks later, behavioral assessments revealed that the mice exhibited excessive digging and self‐grooming activities (Figure 1O1‐P3), similar to those observed in *Nlgn1* KO mice. Furthermore, we utilized AAV viruses expressing Cre driven by the D1 (rAAV‐D1‐CRE) or D2 (rAAV‐D2‐CRE) promoters to suppress NLGN1 expression in D1‐MSNs and D2‐MSNs, respectively. The sample images indicated that these neurons projected to the GPi/SNr and GPe regions, respectively, demonstrating that the D1 and D2 viruses have distinct specificity for direct and indirect pathways (Figure S2A, Supporting Information; Figure 1Q). Behavioral test results showed that the mice in the D2‐MSN KD group exhibited increased self‐grooming and digging time and bouts, while no such increase was observed in the D1‐MSN KD group (Figure 1S1‐T3; Figure S2C–H, Supporting Information). Our previous research has indicated that mice lacking NLGN1 display anxiety and deficits in social memory. To examine the impact of *Nlgn1* KD in D2‐MSN on these behaviors, we conducted open‐field and three‐chamber social behavior tests. As illustrated in Figure S1H–N (Supporting Information), inhibiting NLGN1 in D2‐MSNs did not affect the mice's movement distance, speed, anxiety levels, or social abilities.

### 2.2 NLGN1 in the Dorsal Striatum D2‐MSN is Necessary and Sufficient for Normal Repetitive Behavior

**Figure 2:**
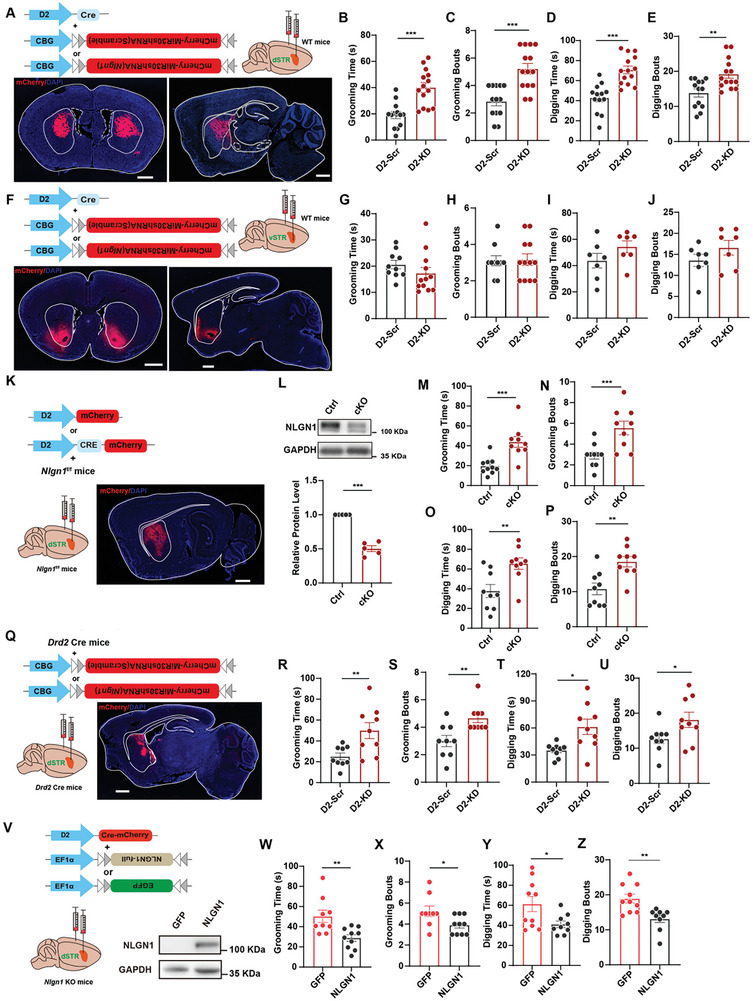
D2‐MSN NLGN1 tunes RRBs. A) Illustration and sample images showing D2‐driven *Nlgn1* KD viruses expression in dorsal striatum. Scale bar: 1mm. B‐C) Statistical graphs depicting self‐grooming time (B) and bouts (C) in mice depicted in panel (A). D‐E) Statistical graphs illustrating digging time (D) and bouts (E) in mice depicted in panel (A). F) Illustration and sample images of D2‐driven *Nlgn1* KD viruses expression in ventral striatum. Scale bar: 1mm. G‐H) Statistical graphs showing self‐grooming time (G) and bouts (H) in mice depicted in panel (F). I‐J) Statistical graphs of digging time (I) and bouts (J) in mice depicted in panel (F). K) Illustration and sample images of D2‐mCherry (Ctrl) or D2‐CRE‐mCherry (cKO) virus expressed in dorsal striatum of *Nlgn1^f/f^* mice. Scale bar: 1mm. L) Representative images (upper) and statistical graph (lower) of NLGN1 protein in striatal tissues from *Nlgn1^f/f^* mice without (Ctrl) or with Cre (cKO) expression in dorsal D2‐MSNs. M‐N) Statistical graphs of self‐grooming time (M) and bouts (N) in *Nlgn1^f/f^* mice depicted in panel (K). O‐P) Statistical graphs of digging time (O) and bouts (P) in *Nlgn1^f/f^* mice depicted in panel (K). Q) Illustration and sample images of *Nlgn1* KD viruses expression in dorsal striatum of D2‐Cre mice. Scale bar: 1mm. R‐S) Statistical graphs of self‐grooming time (R) and bouts (S) in *Drd2*‐Cre mice depicted in panel (Q). T‐U) Statistical graphs of digging time (T) and bouts (U) in *Drd2*‐Cre mice depicted in panel (Q). V) Illustration and sample Western blot images showing D2‐driven HA‐NLGN1 expression in dorsal striatum of *Nlgn1* KO mice. W‐X) Statistical graphs displaying self‐grooming time (W) and bouts (X) in *Nlgn1* KO mice with GFP and HA‐NLGN1 viruses expressed in dorsal D2‐MSNs. Y‐Z) Statistical graphs of digging time (Y) and bouts (Z) in *Nlgn1* KO mice with GFP and HA‐NLGN1 viruses expressed in dorsal D2‐MSNs. Data represent mean ± SEM; Two‐tailed *t*‐test for panel B‐E, G‐J, L‐O, Q‐U and W‐Z; For all the panels, dots represent individual mice. *p < 0.05; **p < 0.01; ***p < 0.001. Also see Figure S3, Tables S1,S2 (Supporting Information).

The striatum is anatomically divided into the dorsal and ventral parts, also known as the neostriatum and nucleus accumbens, which differ in their neural circuits and functions.^[^ ^5^ ^]^ To identify the specific brain region where NLGN1 regulates repetitive behaviors, we suppressed NLGN1 expression in the dorsal and ventral striatal D2‐MSNs, respectively (**Figure** **2**A,F). The results demonstrated that inhibiting NLGN1 expression in the dorsal D2‐MSNs, but not in the ventral D2‐MSNs, increased both the total time and frequency of both RRBs (Figure 2A–J; Figure S3A–D, Supporting Information).

To further confirm that NLGN1 regulates repetitive behaviors through D2‐MSNs in the dorsal striatum, we bilaterally injected the rAAV‐D2‐CRE‐mCherry virus into the dorsal striatum of *Nlgn1^f/f^* mice and allowed five weeks for the deletion of NLGN1 expression in D2‐MSNs of the dorsal striatum (Figure 2K,L). As shown in Figure 2M–P, the mice exhibited a significant increase in self‐grooming and digging time, as well as a higher number of bouts, while maintaining a normal duration of behavior (Figure S3E,F, Supporting Information). We obtained similar results by knocking down NLGN1 in specific D2‐MSNs of *Drd2*‐Cre mice (^[^ ^26^ ^]^; Figure 2Q–U; Figure S3G,H, Supporting Information). In contrast, inhibiting NLGN1 expression in specific D1‐MSNs of *Drd1*‐Cre mice did not lead to any changes in these two repetitive behaviors (Figure S2I–O, Supporting Information).

To confirm that the excessive RRBs were attributable to the absence of NLGN1 in D2‐MSN, we reintroduced *Nlgn1* complementary DNA (cDNA) into the dorsal D2‐MSN by co‐injecting AAV viruses containing D2‐Cre (rAAV‐D2‐CRE‐mCherry) and Cre‐dependent HA‐tagged NLGN1 (rAAV‐EF1α‐DIO‐HA‐NLGN1) into adult *Nlgn1* KO brains (Figure 2V). One week later, we conducted behavioral and protein assays. As illustrated in Figure 2W–Z, the reintroduction of *Nlgn1* effectively reduced both self‐grooming and digging behaviors in KO mice, indicating that NLGN1 in the D2‐MSN of the dorsal striatum is essential for normal repetitive behaviors.

### 2.3 Increased Excitability of D2‐MSN in Mice Lacking NLGN1

**Figure 3:**
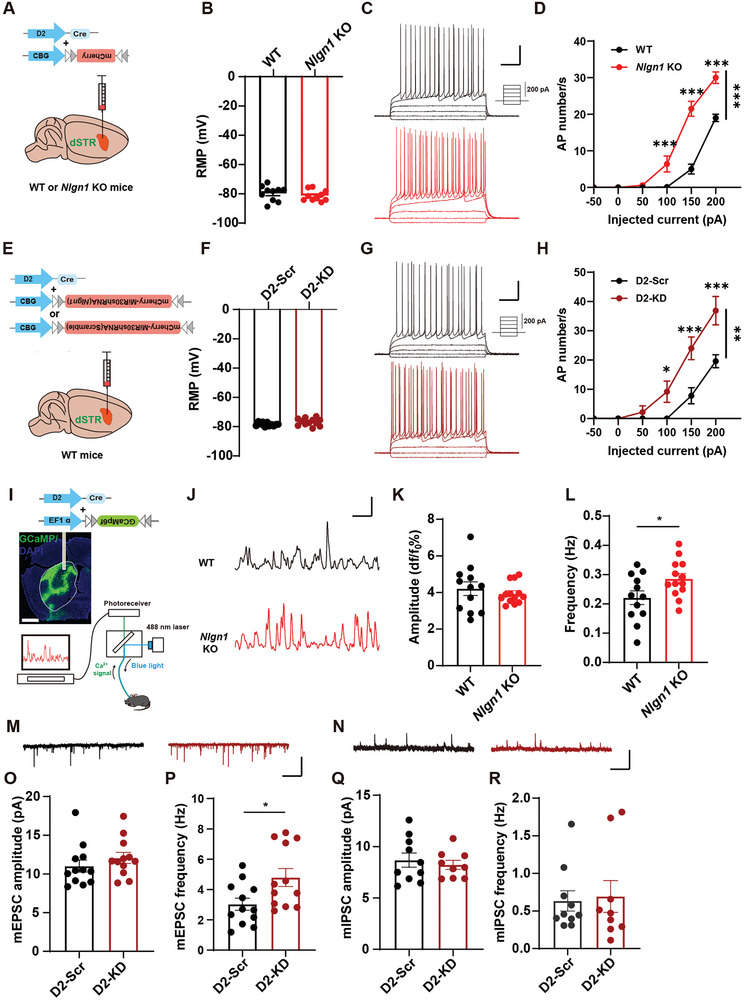
NLGN1 deficiency enhances D2‐MSN excitability. A) Illustration of D2‐driven mCherry expression in dorsal striatum. B) Statistical graph of resting membrane potentials of D2‐MSNs from WT and *Nlgn1* KO mice. C) Current injection intensity illustration and sample traces of injecting current evoked action potentials in D2‐MSNs from WT and *Nlgn1* KO mice. Scale bar: 0.2 s/25 mV. D) Statistical curves of injecting currents evoked action potential numbers in D2‐MSNs from WT and *Nlgn1* KO mice. E) Illustration of D2‐driven scramble or *Nlgn1* KD viruses expression in dorsal striatum of WT mice. F) Statistical graph of resting membrane potentials of D2‐MSNs from WT and *Nlgn1* KO mice. G) Current injection intensity illustration and sample traces of injecting current evoked action potentials in D2‐MSNs from WT and *Nlgn1* KD mice. Scale bar: 0.2 s/25 mV. H) Statistical curves of injecting currents evoked action potential numbers in D2‐MSNs from WT and *Nlgn1* KD mice. I) Illustration and sample image of in vivo optic fiber recording in WT and *Nlgn1* KO mice. J) Sample traces of Ca^2+^ signals of dorsal striatal D2‐MSNs from WT and *Nlgn1* KO mice. Scale bar: 4s/5% df/f_0_. K) Statistical graph of the amplitude of Ca^2+^ events of dorsal striatal D2‐MSNs from WT and *Nlgn1* KO mice. L) Statistical graph of the frequency of Ca^2+^ events of dorsal striatal D2‐MSNs from WT and *Nlgn1* KO mice. M‐N) Sample traces of mEPSC (M) and mIPSC (N) of dorsal striatal D2‐MSNs from WT and *Nlgn1* KD mice. Scale bar: 2 s/20 pA. O‐P) Statistical graphs of the mEPSC amplitude (O) and frequency (P) of dorsal striatal D2‐MSNs from WT and *Nlgn1* KD mice. Q‐R) Statistical graphs of the mIPSC amplitude (Q) and frequency (R) of dorsal striatal D2‐MSNs from WT and *Nlgn1* KD mice. Data represent mean ± SEM; Two‐tailed *t*‐test for panel B, F, K, L and O‐R, repeated two‐way ANOVA with Fisher's LSD post hoc test for panel D and H; For panel B, F and O‐R, dots represent individual cells, for panel K and L, dots represent individual mice. *p < 0.05; **p < 0.01; ***p < 0.001. Also see Figure S4 and Tables S1,S2 (Supporting Information).

To explore the cellular mechanisms by which NLGN1 regulates RRBs, we initially labeled D2‐MSN with D2‐driven Cre and Cre‐dependent mCherry viruses (rAAV‐D2‐CRE and rAAV‐DIO‐mCherry, **Figure** **3**A). We then assessed the excitability of D2‐MSNs in the dorsal striatum of *Nlgn1* KO and WT mice using whole‐cell clamp recordings. The results indicated no significant alterations in the resting membrane potential (RMP) following *Nlgn1* deletion (Figure 3B; Table S3, Supporting Information). However, D2‐MSN excitability was significantly higher in *Nlgn1* KO mice compared to WT mice. (Figure 3C,D). We further confirmed these findings in D2‐MSNs of *Nlgn1* KD mice (Figure 3E–H). We further analyzed the electrophysiological characteristics and voltage‐dependent sodium and potassium currents of NLGN1 KO D2‐MSNs (Table S3 and Figure S4, Supporting Information). The results indicated that D2‐MSNs with NLGN1 deficiency exhibited lower after‐hyperpolarization potential (AHP) (Figure S4A,B, Supporting Information) and faster action potential (AP) rise time (Table S3, Supporting Information), while displaying larger voltage‐dependent sodium current (Figure S4C,D, Supporting Information) and smaller voltage‐dependent potassium current (Figure S4E,F, Supporting Information). These findings suggest that the heightened activity of *Nlgn1*‐deficient D2‐MSNs may be associated with abnormal functions of voltage‐gated ion channels.

To investigate whether D2‐MSNs are also hyperexcited in living *Nlgn1*‐deficient animals, we conducted in vivo fiber photometry recordings to measure the Ca^2+^ signals of D2‐MSNs in the dorsal striatum of WT and *Nlgn1* KO mice (Figure 3I). As shown in Figure 3J–L, the frequency of Ca^2+^ events was higher in *Nlgn1* KO D2‐MSNs compared to WT mice, while the amplitude remained constant. These results confirm that the excitability of D2‐MSNs is increased in *Nlgn1* KO mice both in vitro and in vivo. Additionally, we demonstrated that there were no significant differences in the characteristics of D2‐MSN calcium events between WT and *Nlgn1* KO mice (Figure S4I–K, Supporting Information).

Previous studies have demonstrated that abnormal synaptic transmission may be linked to the generation of RRBs.^[^ ^13^, ^27^, ^28^ ^]^ We conducted recordings of miniature excitatory postsynaptic current (mEPSC) and miniature inhibitory postsynaptic current (mIPSC) in D2 medium spiny neurons (MSNs). The results indicated that D2‐MSNs with *Nlgn1* supression via virus exhibited a significantly increased frequency of mEPSC, while the amplitude of mEPSC and both the amplitude and frequency of mIPSC remained normal (Figure 3M–R). This suggests an enhancement of excitatory synaptic input in *Nlgn1*‐deficient D2‐MSNs. To investigate whether enhanced glutamatergic synaptic transmission is the cause of increased neural excitability in *Nlgn1*‐deficient D2‐MSNs, we utilized ionotropic glutamate receptor inhibitors NBQX (20 µM) and AP5 (50 µM) to perfuse brain slices and block AMPA and NMDA receptors. Through the analysis of D2‐MSN excitability, we found that the perfusion of NBQX and AP5 did not diminish the abnormal excitation of *Nlgn1* KO D2‐MSNs when compared to WT control neurons (Figure S4L,M, Supporting Information). Furthermore, we bilaterally injected NBQX (100 µM) and AP5 (250 µM) into the dorsal striatum of *Nlgn1* KO and WT mice via cannulas and performed behavioral tests along with fiber photometry calcium signal detection. The results showed that these two ionotropic glutamate receptors in the striatum reduced the frequency, but not the amplitude of D2‐MSN Ca^2+^ events (Figure S4Q–V, Supporting Information), and blurred the difference in self‐grooming time between WT and *Nlgn1* KO mice, while having no effect on the difference in digging time (Figure S4N,O, Supporting Information). These findings suggest that the increased excitability of D2‐MSNs and the RRBs, particularly excessive digging observed in *Nlgn1* KO mice, are not primarily driven by heightened glutamatergic synaptic transmission.

### 2.4 The Hyperexcited D2‐MSN Promotes RRBs

**Figure 4:**
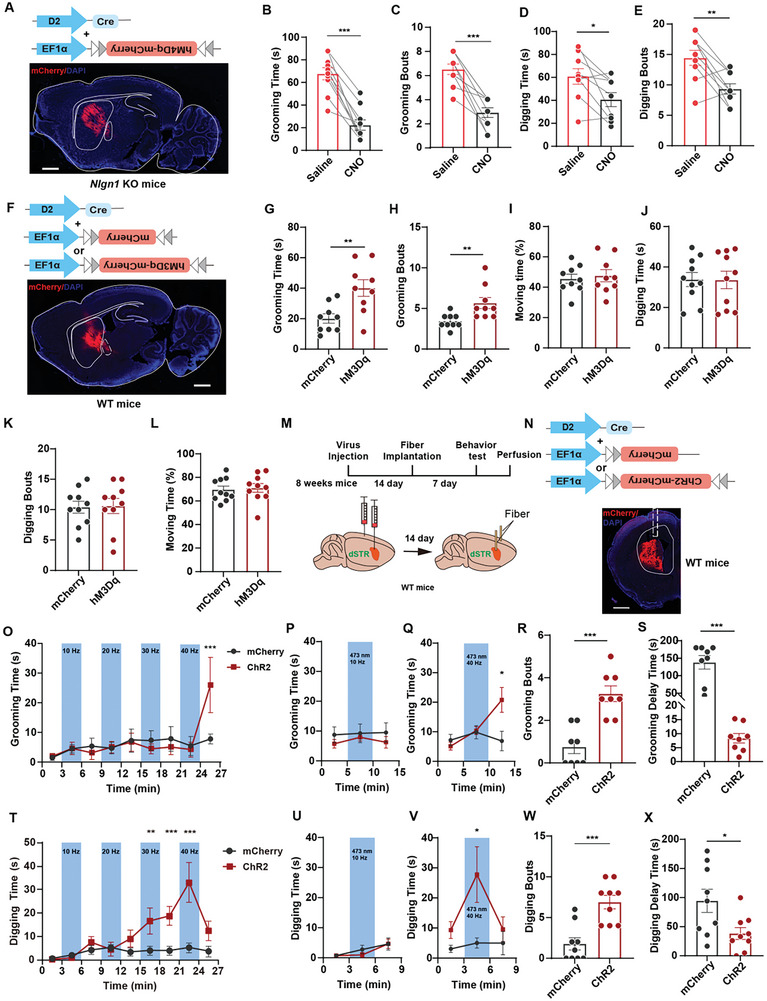
Activating dorsal striatal D2‐MSN promotes RRBs. A) Illustration and sample image of D2‐driven hM4d(Gi)‐mCherry expression in dorsal striatum of *Nlgn1* KO mice. Scale bar: 1 mm. B‐C) Statistical graphs of self‐grooming time (B) and bouts (C) in saline and CNO‐treated dorsal striatal D2‐MSN hM4d(Gi) expressed *Nlgn1* KO mice. D‐E) Statistical graphs of digging time (D) and bouts (E) in saline and CNO‐treated dorsal striatal D2‐MSN hM4d(Gi) expressed *Nlgn1* KO mice. F) Illustration and sample image of D2‐driven hM3d(Gq)‐mCherry expression in dorsal striatum of WT mice. Scale bar: 1mm. G‐I) Statistical graphs of self‐grooming time (G), bouts (H) and moving time percentage (I) in saline and CNO (0.3 mg kg^−1^) treated dorsal striatal D2‐MSN hM3d(Gq) expressed WT mice. J‐L) Statistical graphs of digging time (J), bouts (K) and moving time percentage (L) in saline and CNO (0.3 mg kg^−1^) treated dorsal striatal D2‐MSN hM3d(Gq) expressed WT mice. M‐N) Illustration and sample images of D2‐driven ChR2‐mCherry expression in dorsal striatum of WT mice. The dashed box marking the location of the implanted fiber. Scale bar: 1mm. O) Statistical curves of self‐grooming time of mCherry and ChR2 expressed WT mice under different frequencies of 473 nm blue light stimulation. P‐Q) Statistical curves of self‐grooming time of mCherry and ChR2 expressed WT mice under 10 Hz (P) and 40 Hz (Q) of blue light stimulation. R) Statistical graph of grooming bouts of mCherry and ChR2 expressed WT mice after 40 Hz stimulation in panel (Q). S) Statistical graph of grooming delay time of mCherry and ChR2 expressed WT mice after 40 Hz stimulation in panel (Q). T) Statistical curves of digging time of mCherry and ChR2 expressed WT mice under different frequencies of 473nm blue light stimulation. U‐V) Statistical curves of digging time of mCherry and ChR2 expressed WT mice under 10 Hz (U) and 40 Hz (V) of blue light stimulation. W) Statistical graph of digging bouts of mCherry and ChR2 expressed WT mice during 40 Hz stimulation in panel (V). X) Statistical graph of digging delay time of mCherry and ChR2 expressed WT mice during 40 Hz stimulation in panel (V). Data represent mean ± SEM; Two‐tailed paired *t*‐test for panel B‐E, two‐tailed unpaired *t*‐test for panel G‐L, R‐S and W‐X, repeated two‐way ANOVA with Fisher's LSD post hoc test for panel O‐Q and T‐V; For all the panels, dots represent individual mice. *p < 0.05; **p < 0.01; ***p < 0.001. Also see Figures S5,S6 and Tables S1,S2 (Supporting Information).

To determine whether abnormal activation of D2‐MSNs is essential for the excessive RRBs in *Nlgn1* KO mice. We utilized the designer receptors exclusively activated by designer drugs (DREADD) system to suppress D2‐MSN activity. We bilaterally injected AAV viruses expressing D2‐Cre and floxed engineered M4‐muscarinic receptor (rAAV‐EF1α‐DIO‐hM4D(Gi)‐mCherry) into the dorsal striatum of WT and *Nlgn1* KO mice (**Figure** **4**A; Figure S5A, Supporting Information). To activate hM4D(Gi), clozapine‐N‐oxide (CNO, 1 mg kg^−1^, intraperitoneally) was administered 20 min prior to the behavioral tests. Activation of hM4D(Gi) in *Nlgn1* KO mice, but not in WT mice, significantly reduced the duration and frequency of self‐grooming and digging behaviors (Figure 4B–E; Figure S5B,C, Supporting Information).

Next, we investigated whether increased activity of D2‐MSNs in WT mice could promote RRBs. We bilaterally injected AAV viruses expressing D2‐Cre and floxed engineered M3‐muscarinic receptor (rAAV‐EF1α‐DIO‐hM3D(Gq)‐mCherry) into the dorsal striatum of WT mice (Figure 4F) and conducted self‐grooming and digging tests two weeks later. Considering that activating D2‐MSN with a high dose of CNO may inhibit animal movement,^[^ ^27^ ^]^ we initially administered a low dose (0.3 mg kg^−1^) of CNO and performed behavioral tests 20 min later. The results indicated that the hM3D(Gq)‐mCherry‐expressing mice exhibited an increase in both the duration and frequency of self‐grooming, while their moving and digging times remained normal (Figure 4G–L; Figure S5F,G, Supporting Information). To determine if the observed behaviors were related to incomplete activation of hM3D(Gq), we increased the CNO dose to 1 mg kg^−1^. The results showed a significant reduction in the moving time of the hM3D(Gq)‐expressing mice, accompanied by increased self‐grooming time, while digging time remained intact (Figure S5H–N, Supporting Information). These findings suggest that distinct cellular mechanisms underlie these two types of RRBs. Activation of the Gq signaling pathway is sufficient to promote self‐grooming behavior, but not digging behavior.

To investigate whether the generation of RRBs is influenced by the specific firing pattern of D2‐MSNs, we bilaterally expressed the optogenetic activation element ChR2 in the D2‐MSNs of WT mice using viral vectors (rAAV‐D2‐CRE and rAAV‐EF1α‐DIO‐hChR2(H134R)‐ mCherry). Optical fibers were implanted two weeks after the viral injection and one week prior to the behavioral tests. Initially, we delivered varying frequencies of 473 nm blue light stimulation at 4 mW and conducted self‐grooming tests (Figure 4M,N; Figure S6A, Supporting Information). As shown in Figure 4O, none of the light stimuli at 10 Hz, 20 Hz, 30 Hz, or 40 Hz promoted self‐grooming behavior during the light delivery; however, the mice exhibited excessive self‐grooming behavior following the 40 Hz light stimulation. To eliminate the impact of continuous stimuli, we administered 40 Hz stimulation to a separate group of D2‐MSN ChR2‐expressing mice, with 10 Hz stimulation serving as the control. The results confirmed that after the completion of the 40 Hz stimulation, the mice displayed increased self‐grooming time and bouts, but did not show any increase during the stimulation (Figure 4P–R). Furthermore, we observed that the mice rapidly began self‐grooming ≈10 s after the 40 Hz light stimulation, significantly reducing the delay time for the presentation of grooming behavior (Figure 4S). These experiments suggest a possible relationship that the generation of self‐grooming behavior in mice is associated with the high‐frequency firing and subsequent decrease in activity of D2‐MSNs.

Next, we delivered light stimulation at various frequencies and conducted digging behavior tests in a cage with bedding. The results showed that mice exhibited significantly increased digging behavior during and after exposure to 30 Hz and 40 Hz light stimulation (Figure 4T). We also administered 10 Hz and 40 Hz light stimuli using independent mice expressing D2‐MSN ChR2. The findings indicated that the mice displayed increased digging time and bouts during and after high‐frequency stimulation (40 Hz) (Figure 4U–W). Additionally, the latency of digging behavior was significantly reduced under 40 Hz light stimulation (Figure 4X). A previous study has demonstrated that high‐intensity and high‐frequency light activation of D2‐MSN can decrease the movement time of mice.^[^ ^29^ ^]^ We analyzed the proportion of time that mice spent moving during and after 10 Hz and 40 Hz light stimulation in two different scenarios and found no significant difference compared to the control group (Figure S6B–I, Supporting Information), suggesting that the stimulation paradigms we employed did not impair locomotion.

In summary, we found that inhibiting the activity of D2‐MSN in *Nlgn1* KO mice reduced the duration of both types of RRBs. Activating D2‐MSN in WT mice stimulated the generation of RRB; however, the activation patterns required for self‐grooming and digging differed.

### 2.5 RRBs are Positively Correlated with the Neural Activity of D2‐MSN

**Figure 5:**
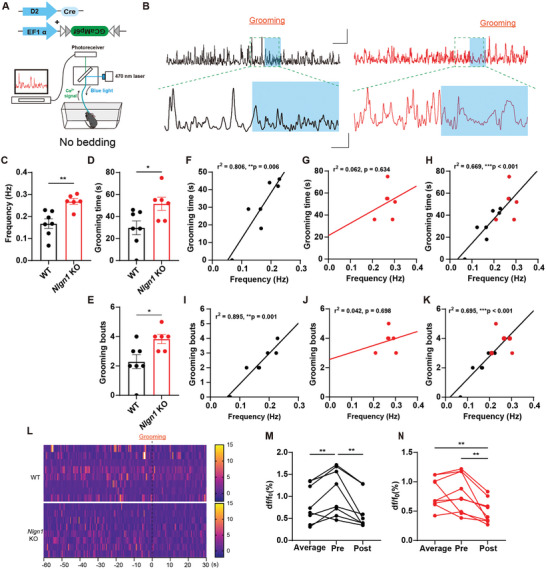
Optic fiber recording of Ca^2+^ signals of self‐grooming behavior. A) Illustration of in vivo optic fiber recording of mice in a cage without bedding. B) Sample traces of Ca^2+^ signals of WT (left) and *Nlgn1* KO (right) dorsal striatal D2‐MSN during self‐grooming. Scale bar: 15s/4% df/f_0_ (up) and 3s/2% df/f_0_ (below). C) Statistical graph of the Ca^2+^ event frequency of WT and *Nlgn1* KO dorsal striatal D2‐MSN. D) Statistical graph of self‐grooming time of WT and *Nlgn1* KO mice during recording. E) Statistical graph of self‐grooming bouts of WT and *Nlgn1* KO mice during recording. F‐H) Correlation matrix analysis of grooming time and Ca^2+^ event frequency in WT (F), *Nlgn1* KO (G) and combined (H) groups. I‐K) Correlation matrix analysis of grooming bouts and Ca^2+^ event frequency in WT (I), *Nlgn1* KO (J) and combined (K) groups. L) Heat maps of WT and *Nlgn1* KO D2‐MSN Ca^2+^ signals around grooming behavior. M‐N) Statistical graphs of D2‐MSN Ca^2+^ signal intensity during average conditions, 10s before grooming onset, and 10s after the onset of grooming in WT (M) and *Nlgn1* KO (N) mice. Data represent mean ± SEM; Two‐tailed unpaired *t*‐test for panel C, D and H, correlation matrix analysis for panel E‐G and I‐K, repeated one‐way ANOVA with Fisher's LSD post hoc test for panel M and N; Dots represent individual mice for panel C‐K, and represent behavior bout for panel M‐N. *p < 0.05; **p < 0.01; ***p < 0.001. Also see Table S1 (Supporting Information).

**Figure 6:**
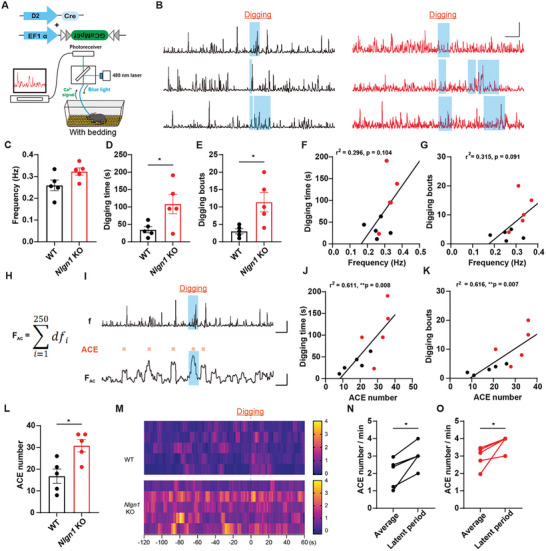
Optic fiber recording of Ca^2+^ signals of digging behavior. A) Illustration of in vivo optic fiber recording of mice in a cage with bedding. B) Sample traces of Ca^2+^ signals of WT (left) and *Nlgn1* KO (right) dorsal striatal D2‐MSN during digging. Scale bar: 15s/4% df/f_0_. C) Statistical graph of the Ca^2+^ event frequency of WT and *Nlgn1* KO dorsal striatal D2‐MSNs. D‐E) Statistical graphs of digging time (D) and bouts (E) of WT and *Nlgn1* KO mice during recording. F‐G) Correlation matrix analysis of digging time (F) and bouts (G) with Ca^2+^ event frequency in combined groups. H‐I) Method (H) and sample trace (I) of Ca^2+^ signal accumulation. Scale bar: 15s/4% df/f_0_ (up) and 15s/10 df/f_0_ (below). J‐K) Correlation matrix analysis of digging time (J) and bouts (K) with ACE number in combined groups. L) Statistical graph of ACE number of WT and *Nlgn1* KO dorsal striatal D2‐MSNs. M) Heat maps of WT and *Nlgn1* KO D2‐MSN Ca^2+^ signals around digging behavior. N‐O) Statistical graphs of D2‐MSN Ca^2+^ signal intensity of average and 60s before digging onset in WT (N) and *Nlgn1* KO (O) mice. Data represent mean ± SEM; Two‐tailed unpaired *t*‐test for panel C‐E and L, correlation matrix analysis for panel F‐G and J‐K, two‐tailed paired *t*‐test for panel N and O; For all the panels, dots represent individual mice. *p < 0.05; **p < 0.01, ***p < 0.001. Also see Figure S7 and Tables S1,S2 (Supporting Information).

To further investigate the role of D2‐MSN activity in generating RRBs, we conducted in vivo fiber photometry recordings of Ca^2+^ signals in the dorsal striatal D2‐MSN population of freely moving WT and *Nlgn1* KO mice in cages with bedding and without bedding (**Figures** **5**A and **6**A).

In the unbedded cage, we allowed the mice to move freely for 10 minutes while recording their dorsal D2‐MSN Ca^2^⁺ activity using optical fibers during this period (Figure 5A,B). As shown in Figure 5C–E, the frequency of D2‐MSN calcium activity, as well as the duration and frequency of self‐grooming bouts in *Nlgn1* KO mice, were significantly higher than those in WT mice. To determine whether there is a correlation between calcium activity and self‐grooming behavior, we conducted a correlation matrix analysis. The results, illustrated in Figure 5F–K, revealed significant positive correlations between the frequency of calcium activity and the duration/bouts of self‐grooming in both the WT and combined groups. However, the correlations were inconsistent in *Nlgn1* KO mice, which may be attributed to their high basal activity and existing constraints or homeostatic regulation within their nervous systems. Overall, it is noteworthy that heightened calcium activity was positively correlated with increased self‐grooming behavior in both groups of mice. As shown in Figure 4S, the mice exhibited self‐grooming behavior ≈10 s after the cessation of 40 Hz stimulation, indicating a time lag of ≈10 s between neuronal activity and self‐grooming behavior. Consequently, we compared the Ca^2^⁺ intensities during the average 10 s before and after the onset of self‐grooming behavior. The results indicated that in WT mice, the Ca^2^⁺ intensity during the pre‐period was higher than that during the post‐period and the average value of the entire recording (Figure 5L,M). In *Nlgn1* KO mice, the Ca^2^⁺ intensity during the pre‐period was significantly higher than that during the post‐period, but not compared to the average intensity (Figure 5L,N). This difference may also be related to the elevated basal calcium activity observed in *Nlgn1* KO mice.

Next, we analyzed the Ca^2^⁺ signals of D2‐MSN in WT and *Nlgn1* KO mice before and after the digging behavior under bedded conditions (Figure 6A,B). As shown in Figure 6C–E, the duration and frequency of digging bouts in *Nlgn1* KO mice were significantly greater than those in WT mice. However, there were no significant correlations between the frequency of Ca^2^⁺ activity in D2‐MSN and the duration or frequency of digging bouts (Figure 6F,G; Figure S7A–D, Supporting Information). Given that the onset of digging behavior was delayed by several seconds during the 30 Hz and 40 Hz light stimuli, we speculated that the initiation of digging behavior might be related to the cumulative effect of heightened D2‐MSN activity. Consequently, we constructed the Ca^2^⁺ signal accumulation curve (Figure 6H,I) and analyzed the correlation between accumulated calcium events (ACE) and digging behavior. The results indicated that the number of ACE in *Nlgn1* KO mice was higher than that in WT mice and was significantly positively correlated with both the duration and frequency of digging behaviors in WT and combined groups of mice (Figure 6J–L; Figure S7E–H, Supporting Information). To investigate the potential role of the cumulative effect of D2‐MSN activity in promoting digging behavior, we defined the 60 s prior to digging as the latency period and observed that the number of ACE during this latency period was higher than the average ACE number throughout the entire recording (Figure 6M–O), suggesting a positive relationship between digging behavior and the accumulation of high D2‐MSN activity.

These results suggest that the generation of both self‐grooming and digging behaviors is correlated with the neural activity of D2‐MSN, with distinct activity patterns underlying each behavior.

### 2.6 NLGN1 in D2‐MSN Regulates Hyperexcitation and RRBs Through Excessive PKC Activity

**Figure 7:**
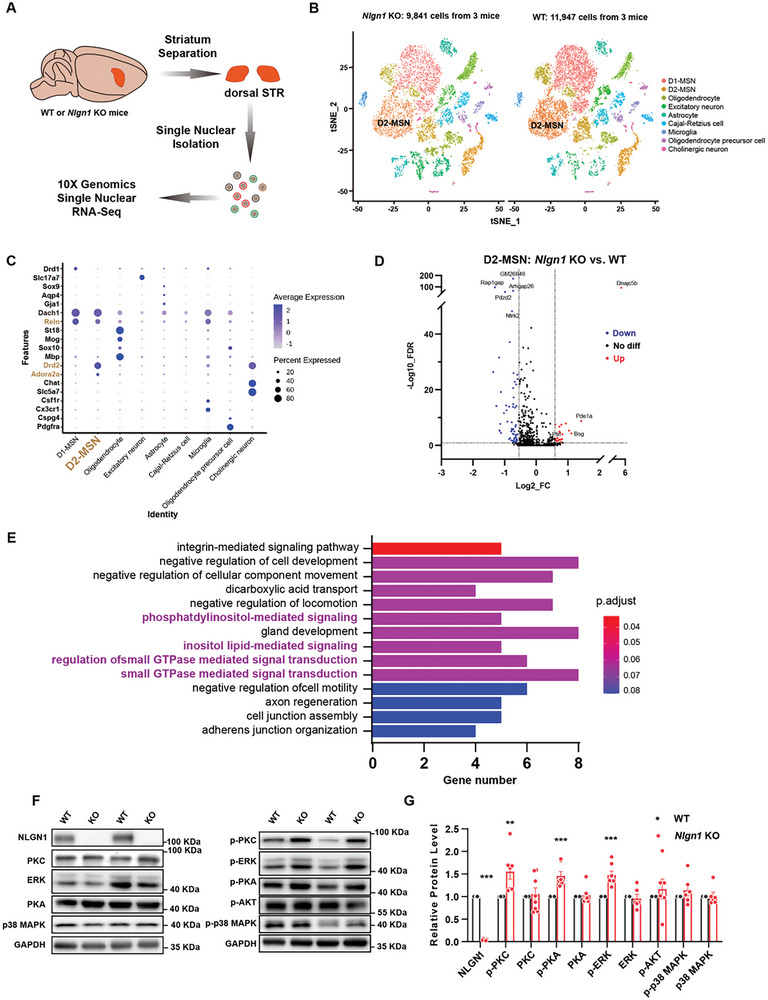
SnRNA‐seq analysis of D2‐MSN in WT and *Nlgn1* KO striatum. A) Schematic diagram illustrating the snRNA‐seq workflow for the dorsal striatum from WT and *Nlgn1* KO mice. B) The t‐SNE visualization of nuclei from *Nlgn1* KO and WT mouse dorsal striatum. Nuclei are color‐coded by cell type. C) Assignment of clusters to specific cell types based on the expression of known gene markers. D) Volcano plot illustrating the differentially expressed genes in *Nlgn1* KO and WT D2‐MSN. E) Gene ontology pathway enrichment analysis of DEGs in D2‐MSNs highlights phosphatidylinositol‐mediated signaling and small GTPase‐mediated signal transduction (highlighted in purple). F‐G) Representative western blot images (F) and quantification (G) of changes in phosphorylated protein levels in the striatal tissues of WT and *Nlgn1* KO mice. Data represent mean ± SEM; Two‐tailed unpaired *t*‐test for panel G; For panel B, dots represent individual cells, for panel D, dots represent individual genes, for panel G, dots represent individual mice. *p < 0.05; **p < 0.01, ***p < 0.001. Also see Figure S8 and Tables S1,S2 (Supporting Information).

To investigate the molecular mechanisms by which NLGN1 modulates RRBs and D2‐MSN hyperexcitation, we isolated dorsal striatum tissues of WT and *Nlgn1* KO mice and performed single‐nucleus RNA sequencing (snRNA‐seq, **Figure** **7**A). To visualize and identify cell populations with distinct transcriptional signatures, we conducted a nonlinear dimensionality reduction (t‐distributed stochastic neighbor embedding (t‐SNE)) on a total of 21 788 cells (11,947 from WT and 9,841 from *Nlgn1* KO) derived from pooled tissues (n = 3) (Figure 7B). We then utilized a list of established marker genes to assign cluster identities (Figure 7C), including a cluster that exhibited high expression of *Drd2*, *Reln*, and *Adora2a*, which we designated as D2‐MSN.^[^ ^30^, ^31^, ^32^ ^]^ Subsequently, we measured the differentially expressed genes (DEGs) between WT and *Nlgn1* KO D2‐MSN and identified 56 downregulated genes and 21 upregulated genes in the *Nlgn1* KO group based on the following criteria: fold change > 1.5 and False Discovery Rate (FDR) < 0.1 (Table S4, Supporting Information). Gene ontology (GO) analyses of the DEGs highlighted several biological processes related to neuronal activity, including phosphatidylinositol‐mediated signaling and small GTPase‐mediated signal transduction (Figure 7E).

**Figure 8:**
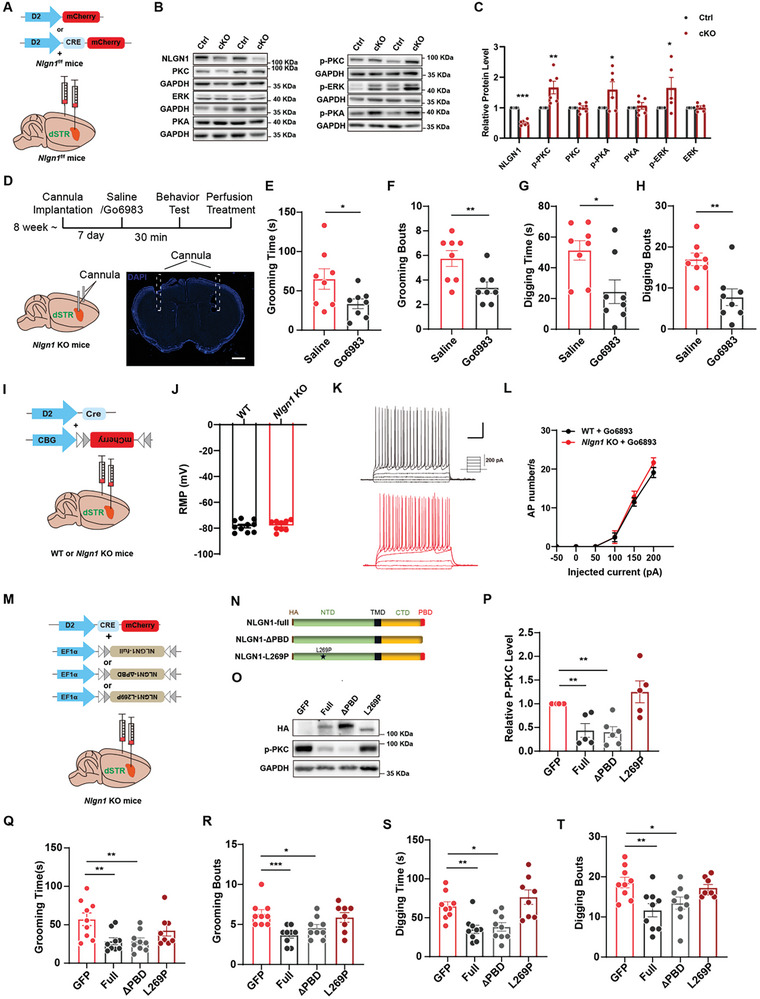
PKC signaling is involved in the regulation of NLGN1 on D2‐MSN activity and RRBs. A) Illustration of D2‐mCherry (Ctrl) or D2‐CRE‐mCherry (cKO) virus expressed in dorsal striatum of *Nlgn1^f/f^* mice. B‐C) Sample images (B) and statistical graph (C) of protein expression in striatum from two groups of mice depicted in panel A. D) Illustration and sample image of cannula implantation and drug injection in *Nlgn1* KO dorsal striatum. The cannula implantation site is highlighted by the dashed box. Scale bar: 1mm. E‐F) Statistical graphs of self‐grooming time (E) and bouts (F) in saline and Go6983 injected *Nlgn1* KO mice. G‐H) Statistical graphs of digging time (G) and bouts (H) in saline and Go6983 injected *Nlgn1* KO mice. I) Illustration of D2‐driven mCherry expression in dorsal striatum. J) Statistical graph of resting membrane potentials of Go6983 treated WT and *Nlgn1* KO D2‐MSN. K) Current injection intensity illustration and sample traces of injecting current evoked action potentials in Go6983 treated WT and *Nlgn1* KO D2‐MSN. Scale bar: 0.2s/25 mV. L) Statistical curves of injecting currents evoked action potential numbers in Go6983 treated WT and *Nlgn1* KO D2‐MSN. M‐N) Schematic diagram (M) of the viral injection strategy in *Nlgn1* KO mice and illustrations (N) of various forms of NLGN1 protein. O‐P) Sample images (O) and statistical graph (P) of HA and p‐PKC levels in different viruses expressed *Nlgn1* KO striatal tissues. Q‐R) Statistical graphs of self‐grooming time (Q) and bouts (R) in striatal D2‐driven GFP, NLGN1‐full, NLGN1‐ΔPBD and NLGN1‐L269P viruses expressed *Nlgn1* KO mice. S‐T) Statistical graphs of digging time (S) and bouts (T) in striatal D2‐driven GFP, NLGN1‐full, NLGN1‐ΔPBD and NLGN1‐L269P viruses expressed *Nlgn1* KO mice. Data represent mean ± SEM; Two‐tailed unpaired *t*‐test for panel C, E‐H and J, repeated two‐way ANOVA with Fisher's LSD post hoc test for panel L, one‐way ANOVA test with LSD post hoc multiple comparisons for panel P‐T; For panel C, E‐H and P‐T, dots represent individual mice, for panel J, dots represent individual cells. *p < 0.05; **p < 0.01, ***p < 0.001. Also see Figure S8 and Tables S1,S2 (Supporting Information).

Therefore, we hypothesize that the activity of specific protein kinases involved in phosphatidylinositol metabolism and the small GTPase pathway may be altered.^[^ ^33^, ^34^, ^35^ ^]^ This alteration could further impact the cellular activity of *Nlgn1*‐deficient D2‐MSNs and lead to the generation of excessive RRBs. Subsequently, we measured the phosphorylation levels of protein kinase C (PKC), protein kinase A (PKA), protein kinase B (Akt), p38 mitogen‐activated protein kinase (p38‐MAPK), and extracellular signal‐regulated kinase (ERK) in the *Nlgn1* KO and WT striatal tissues. The results indicated that the activities of PKC, PKA, and ERK significantly increased, while the levels of Akt and p38‐MAPK remained unchanged after *Nlgn1* deletion (Figure 7F,G). We obtained similar findings in D2‐MSN *Nlgn1* cKO mice (**Figure** **8**A–C), confirming that the increased activities of PKC, PKA, and ERK in D2‐MSNs were a consequence of NLGN1 deficiency. Additionally, we examined other synaptic proteins and receptors known to be expressed in D2‐MSNs and involved in synaptic and neuronal activity.^[^ ^6^, ^36^, ^37^ ^]^ The protein levels of NLGN2 and NLGN3, dopamine receptors D1 and D2, as well as ionotropic glutamate receptors GluA1, GluA2, NR1, NR2A, and NR2B, remained unchanged (Figure S8A,B, Supporting Information).

Next, we assessed the impact of activated kinases on RRBs in *Nlgn1* KO mice using pharmacological methods. As illustrated in Figure 8D and Figure S8C (Supporting Information), we bilaterally injected inhibitors (Go6983 for PKC, H89 for PKA, and AG126 for ERK) into the dorsal striatum of both *Nlgn1* KO and WT mice via cannulas. The results indicated that only the PKC inhibitor Go6983 significantly reduced self‐grooming and digging time, as well as the number of bouts in *Nlgn1* KO mice, while having no effect on WT mice (Figure 8D–H; Figure S8H–K, Supporting Information). The PKA inhibitor H89 diminished the digging phenotype but had less effect on excessive grooming behavior in *Nlgn1* KO mice. The ERK inhibitor AG126 did not reduce any excessive RRBs in *Nlgn1* KO mice (Figure S8C–G, Supporting Information). These findings suggest that excessive PKC activity may be the molecular mechanism underlying the increased RRBs in *Nlgn1* KO mice. To investigate whether this is also linked to heightened activity following *Nlgn1* deficiency, we conducted whole‐cell patch‐clamp recordings. As shown in Figure 8I–L, the activity of D2 MSNs in *Nlgn1* KO slices decreased to WT level after perfusion with the PKC inhibitor Go6983. We further examined the effects of the PKC inhibitor on the electrophysiological characteristics and calcium activity of *N* *lgn1* KO D2‐MSN. The results showed that Go6983 eliminated the differences in voltage‐gated potassium current and AHP between *Nlgn1* KO and WT D2‐MSNs, but had no effect on voltage‐gated sodium current (Figure S9A–F, Supporting Information). Fiber photometry calcium imaging experiments conducted before and after drug injection showed that Go6983 significantly inhibited both the amplitude and frequency of Ca^2+^ events in *Nlgn1* KO D2‐MSN (Figure S9G–I, Supporting Information). All of these results suggest an effective regulatory effect of PKC on D2‐MSN activity.

Finally, we would like to investigate the mechanism by which NLGN1 regulates PKC activity and RRBs. We expressed full‐length NLGN1 (NLGN1‐FL) and a variant of NLGN1 lacking the PDZ binding domain (NLGN1‐ΔPBD), as well as a known ASD‐associated point mutation of NLGN1 (NLGN1‐269), which has been reported to be unable to translocate to the cell membrane,^[^ ^38^ ^]^ in the dorsal D2‐MSN of *Nlgn1* KO mice (Figure 8M–O). The results indicated that both NLGN1‐FL and NLGN1‐ΔPBD effectively inhibited the elevated phosphorylation level of PKC and reduced the duration and frequency of self‐grooming and digging behaviors in *Nlgn1* KO mice, whereas NLGN1‐269 did not (Figure 8O–T; Figure S8L,M, Supporting Information). This suggests that the regulation of PKC activity and RRBs by NLGN1 may not be related to its PBD, but rather depends on its normal trafficking or extracellular domains.

## 3 Discussion

Excessive RRBs were hallmark symptoms of ASD, and their underlying mechanisms remain elusive. In this study, we found that the absence of the ASD‐associated molecule NLGN1 in dorsal striatum D2‐MSN resulted in excessive self‐grooming and digging behaviors. The generation of these two RRBs depended on increased activity of D2‐MSN, albeit with different patterns of activity. Furthermore, we discovered that heightened PKC activity in D2‐MSN was a crucial factor contributing to the hyperexcitability of D2‐MSN and the manifestation of RRBs in *Nlgn1*‐deficient mice.

To identify the key brain regions and neurons influenced by NLGN1 in RRB, we initially targeted multiple brain regions by suppressing *Nlgn1*. The results indicate that inhibiting *Nlgn1* specifically in the D2‐MSNs of the dorsal striatum resulted in excessive RRBs in *Nlgn1*‐deficient mice, characterized by increased self‐grooming and digging time, as well as the frequency of bouts, while bout duration remained intact (Figure 2A–Q). Furthermore, overexpressing NLGN1 or NLGN1‐ΔPBD in the dorsal D2‐MSN of *Nlgn1* KO mice reversed their excessive repetitive behaviors (Figure 2V–Z and Figure 8M–T), indicating that NLGN1 regulates the production of RRBs through the dorsal striatum D2‐MSN. These findings are consistent with previous reports linking D2‐MSN dysfunction to excessive self‐grooming behavior.^[^ ^27^, ^39^, ^40^, ^41^ ^]^ Brain regions such as CeA, PVH, mPFC and OFC have been reported to be involved in the regulation of self‐grooming behavior.^[^ ^8^, ^10^ ^]^ Additionally, studies have demonstrated the involvement of the direct pathway within the striatum, as well as D3 neurons in the ventral striatum, in self‐grooming behavior.^[^ ^42^, ^43^, ^44^ ^]^ These findings suggest that typical repetitive behaviors require the coordination of various brain regions and neural circuits, each contributing uniquely. In D2‐MSN, the absence of NLGN1 disrupted the balance and synergy between these pathways, resulting in abnormal repetitive behaviors.

The neuronal activity that evokes neurotransmitter release is the primary form of neuronal output. Therefore, we focused on how NLGN1 deficiency influences the activity of D2‐MSNs. First, through whole‐cell patch clamp recordings, we found that the excitability of *Nlgn1*‐deficient D2‐MSNs was significantly increased (Figure 3A–H). Similarly, in vivo recordings demonstrated that the frequency of Ca^2+^ signals was higher in *Nlgn1*‐deficient D2‐MSNs compared to that in WT mice (Figure 3I–L). These findings indicate that NLGN1 deficiency leads to an increase in D2‐MSN excitability, which is consistent with previously reported phenomena in *Shank3*‐deficient mice.^[^ ^27^ ^]^ To investigate whether the overactivation of D2‐MSNs is associated with excessive RRBs, we employed chemogenetic methods to suppress the activity of D2‐MSNs in *Nlgn1* KO mice, resulting in significant reductions in both types of excessive RRBs (Figure 4A–E). Conversely, we induced excessive self‐grooming and digging behaviors in WT mice through chemogenetic or optogenetic activation (Figure 4F–X). Furthermore, we detected positive correlations between high‐frequency D2‐MSN calcium activity and RRBs through in vivo opto‐fiber recordings (Figures 5 and 6), indicating that excessive activation of D2‐MSNs promotes the occurrence of repetitive behaviors. These results align with previous reports that inhibiting the activity of D2‐MSNs in *Sapap3* KO mice reduces their excessive self‐grooming time.^[^ ^39^, ^40^, ^41^, ^45^ ^]^ A body of research links reduced glutamatergic synaptic input in striatal neurons to the development of repetitive behaviors.^[^ ^7^, ^13^, ^25^, ^46^ ^]^ However, we detected enhanced glutamatergic synaptic input in *Nlgn1* KD D2‐MSN (Figure 3P), and inhibition of AMPA and NMDA receptors in the striatum decreased D2‐MSN activity and weakened the difference in self‐grooming time between WT and *Nlgn1* KO mice (Figure S4L–P, Supporting Information). These conflicting findings suggest that glutamatergic synaptic transmission may serve different functions in specific diseases or mutation models. Besides, the literature indicates that inhibiting astrocyte activity in the striatum reduces the excitability of MSNs and prolongs the average duration of self‐grooming behavior, although it does not affect the frequency of this behavior.^[^ ^47^ ^]^ Based on these studies and our findings, we propose that the varying activity levels of MSNs may influence the distinct characteristics of repetitive behaviors, with the activation of D2‐MSNs being more likely to enhance the potential for generating repetitive behaviors.

Our experiments further indicate that the increases in self‐grooming and digging behavior, both in terms of duration and frequency, were associated with the absence of NLGN1 in D2‐MSN and the consequent increase in neuronal activity; however, the underlying cellular mechanisms vary. In this study, we found that inhibiting the excitability of D2‐MSN in *Nlgn1* KO mice or activating D2‐MSN in WT mice through chemogenetic methods, respectively reduced or increased self‐grooming behavior (Figure 4A–L). Through sn‐RNA seq, we identified changes in gene expression related to phosphoinositol signaling and small GTPase signaling pathways in D2‐MSN following *Nlgn1* deletion. This suggests that the activity of proteins such as PKC and PKA may differ, a finding that was confirmed by our subsequent protein assays (Figure 7 and Figure 8A–C). Additionally, we observed that the PKC inhibitor effectively blocked the excessive grooming behavior in *Nlgn1* KO mice but had no significant effect on WT mice (Figure 8D–H; Figure S8J,K, Supporting Information). This indicates that PKC signaling plays a major role in the excessive self‐grooming behavior observed in *Nlgn1* KO mice, while it does not influence the intrinsic grooming behavior of WT mice. Furthermore, we found that the absence of *Nlgn1* resulted in a reduction of voltage‐gated potassium current, and the inhibition of PKC activity mitigated this difference (Figures S4F and S9F, Supporting Information). Previous studies have demonstrated that the excitability of MSNs is related to the activity of voltage‐gated potassium channel KCNQs, which can be regulated by PKC, particularly PKC‐α.^[^ ^48^, ^49^, ^50^ ^]^ These studies are consistent with our findings and may elucidate how *Nlgn1* regulates the excitability of D2‐MSN by inhibiting PKC activity in our study. It's interesting that we also observed an increase in voltage‐gated sodium current in *Nlgn1* KO D2‐MSNs, but it could not be eliminated by the PKC inhibitor (Figures S4D and S9D, Supporting Information). This suggests that NLG1 may also regulate D2‐MSN activity through alternative pathways besides PKC.

Optogenetic experiments revealed that following a brief delay after 40 Hz light stimulation, the mice promptly exhibited self‐grooming behavior (Figure 4M–S). This finding aligns with results obtained from optical fiber experiments, suggesting that high‐frequency excitation of D2‐MSN promotes the onset of self‐grooming behavior (Figure 5). In contrast, we observed that within three minutes of light stimulation, the mice did not display any increase in self‐grooming behavior (Figure 4O,P). Additionally, D2‐MSN activity decreased after the onset of grooming behavior (Figure 5L–N), indicating that the rapid silencing of D2‐MSN may participate in or accompany the generation of self‐grooming behavior. Regarding digging behavior, we found that both chemogenetic inhibition (via hM4d(Gi) activation) and the application of PKC inhibitor reduced digging time and frequency in *Nlgn1* KO mice (Figure 4D,E and Figure 8G,H). However, activation of hM3d(Gq) in WT mice did not increase digging time (Figure 4J–L; Figure S5L–N, Supporting Information). This suggests that the excessive digging behavior observed in *Nlgn1*‐deficient mice is regulated by the Gi‐GPCR and PKC signaling pathways, while activation of the Gq‐GPCR signaling pathway alone is insufficient to induce digging behavior in WT mice. Through optical activation and fiber optic recording experiments, our results indicate that digging behavior may be related to the cumulative effects of high‐frequency activation of D2‐MSN on the downstream neural circuitry (Figure 6). In summary, our findings suggest that both self‐grooming and digging behaviors are associated with *Nlgn1* deficiency in D2‐MSN, as well as with the overexcitation of D2‐MSN; however, they exhibit distinct activity patterns and downstream effects. The PKC signaling pathway is linked to excessive, rather than intrinsic, self‐grooming behavior and is necessary, though not sufficient, for inducing digging behavior. At the same time, we also realized that the high‐frequency activity of D2‐MSN not only exerts a strong inhibitory effect on the downstream GPe, but also suppresses other local MSNs, and striatal cholinergic interneurons.^[^ ^9^, ^51^ ^]^ Given the role of cholinergic interneurons as “pacemakers” in the striatum, intense activation of D2‐MSNs may lead to an overall inhibition of striatal network activity, while also providing negative feedback to the D2‐MSNs themselves.

Finally, regarding the molecular mechanisms associated with NLGN1, we conducted preliminary research. Our previous studies have demonstrated that NLGN1 plays a crucial role in regulating dendritic spine formation and synaptic plasticity through its intracellular PDZ binding domain.^[^ ^21^ ^]^ In this study, we found that supplementing the PBD‐deleted NLGN1 (NLGN1‐ΔPBD) in the D2‐MSN of *Nlgn1* KO mice was able to reverse the behavioral and PKC activity abnormalities observed in *Nlgn1*‐deficient mice. However, ASD‐related point mutation (NLGN1‐L269P), which is incapable of cell surface trafficking, failed to produce similar outcomes (Figure 8M–T). This suggests that the regulation of PKC activity and RRBs by NLGN1 depends on its synaptic functionality rather than its intracellular domain.

In conclusion, this study elucidates how the ASD‐associated molecule NLGN1 regulates the development of restricted and repetitive behaviors in mice, outlining the cellular mechanisms underlying various repetitive behaviors. We also propose that excessive excitation of the dorsal striatum D2‐MSN may be a key mechanism contributing to the heightened RRBs observed in patients and animal models with ASD. Furthermore, our findings may offer valuable insights for interventions and treatments for ASD.

## 4 Experimental Section

### Mice

The *Nlgn1* knockout (KO) mice, generated through the insertion of a neomycin resistance cassette to replace the first two coding exons of the *Nlgn1* gene, were acquired from the Jackson Laboratory (Stock: 008136). *Drd1*‐Cre (MMRRC Tg(*Drd1*‐cre)EY262Gsat), and *Drd2*‐Cre (MMRRC Tg(*Drd2*‐cre)ER44Gsat) mouse lines^[^ ^26^ ^]^ were originated from MMRRC (Stock: 017264‐UCD and 032108‐UCD). The floxed *Nlgn1* (*Nlgn1^f/f^*) mice were generated by Gempharmatech Co., Ltd (Jiangsu, China), with the strategy of LoxP sites flanking the third exon of the *Nlgn1* gene through CRISPR‐Cas9 technology. Genotyping of *Nlgn1* KO mice was conducted using PCR techniques, following previously described methods.^[^ ^21^ ^]^ Genotyping of D1‐Cre/D2‐Cre mice was followed the protocol on the MMRRC. The mice were housed in cages containing two to five individuals each, maintained on a 12‐hour light/dark cycle (7:00 to 19:00), with ad libitum access to food and water. All experimental procedures were carried out during the light cycle. Experimenters were kept blind to the genotype and treatment of the mice throughout the study and all the mice utilized in the experiment were sex balanced, between two and four months‐old. The care and use of all mice adhered to protocols approved by the Animal Care Committee of Southeast University, Nanjing, China (experiment number: 20211101004).

### Antibodies, Reagents, and Viruses

Primary antibodies include anti‐NLGN1 (1:2000; Cat# 129013, Synaptic System), anti‐NLGN2 (1:2000; Cat# 129203, Synaptic System), anti‐NLGN3 (1:2000; Cat#129103, Synaptic System), anti‐GluA1 (1:2000; Cat# MAB2263, Millipore), anti‐GluA2 (1:2000; Cat# MAB397, Millipore), anti‐NR1 (1:2000; Cat# 5704, Cell Signaling Technology), anti‐NR2A (1:2000; Cat# 4205, Cell Signaling Technology), anti‐NR2B (1:2000; Cat# 14544, Cell Signaling Technology), anti‐HA (1:2000; Cat# 51064‐2‐AP, Proteintech), anti‐Drd1(1:2000; Cat# 17934‐1‐AP, Proteintech), anti‐Drd2 (Cat# 55084‐1‐AP, Proteintech), anti‐PKC(1:2000, Cat# 2056, Cell Signaling Technology), anti‐PKC alpha (phospho T497) (1:2000; Cat# ab76016, Abcam), anti‐PKA C‐α(1:2000; Cat#5842, Cell Signaling Technology), anti‐phospho‐PKA C (Thr197) (1:2000; Cat#5661, Cell Signaling Technology), anti‐Erk1/2 (1:2000; Cat#4695, Cell Signaling Technology), anti‐phospho‐Erk1/2 (Thr202/Tyr204) (1:2000; Cat#4370, Cell Signaling Technology), anti‐p38 MAPK (1:2000; Cat#9212, Cell Signaling Technology), anti‐Phospho‐p38 MAPK (Thr180/Tyr182) (1:2000; Cat#4511,Cell Signaling Technology), anti‐ChAT (1:50; Cat#AB144, Sigma‐Aldrich), anti‐GAPDH (1:4000; Cat# 60004‐1‐Ig, Proteintech). Secondary antibodies included: goat anti‐rabbit (1:2000; Cat# SA00001‐2, Proteintech) and goat anti‐mouse (1:2000; Cat# SA00001‐1, Proteintech).

### Reagents

Reagents include Go6983 (Cat# A8343, APExBIO), H89 (Cat# B2190, APExBIO), AG126 (Cat# C4338, APExBIO), NBQX (Cat#N171, Sigma), Picrotoxin (PTX, Cat# R284556, Sigma), AP5 (Cat# 0106, Tocris), 1.25% Tribromoethanol (Cat# M2920, EasyCheck), Surcose (Cat# A610498, Sangon Biotech), Paraformaldehyde (Cat# A500684, Sangon Biotech), Clozapine N‐oxide (Cat# HY‐17366, MedChemExpress), Saline (Cat# CZ0034, Leagene), PBS (Cat# 311‐010‐CL, Wisent) and Mineral oil (Cat# BS927, Biosharp). All drugs used in this study are commercially available and are solely for use in animal experiments. The specific drug information, including instructions for use, dosage, and precautions, can be found on relevant official websites or professional databases to obtain more detailed data and safety information.

### Viruses

The rAAV‐CMV‐bGlobin‐Flex‐EGFP‐MIR30shRNA (*Nlgn1*) (Cat# Y6552), rAAV‐CBG‐DIO‐mCherry‐MIR30shRNA (*Nlgn1*)‐WPRE (Cat# Y12413), rAAV‐EF1α‐DIO‐HA‐NLGN1‐WPRE (Cat# H16393) viruses were purchased from Obio Technology (Shanghai, China). The sequence of NLGN1‐shRNA used in this paper is: 5′‐GGAAGGTACTGGAAATCTG‐3′,^[^ ^21^, ^52^ ^]^

The rAAV‐hSyn‐CRE‐WPRE (Cat# PT‐0136), rAAV‐VGAT1‐CRE‐mCherry‐bGH (Cat# PT‐0533), rAAV‐VGAT1‐mCherry‐WPRE (Cat# PT‐0325), rAAV‐ChAT‐CRE‐WPRE(Cat# PT‐0607), rAAV‐D1‐CRE‐WPRE (Cat# PT‐0570), rAAV‐D2‐CRE‐WPRE (Cat# PT‐0571), rAAV‐D2‐CRE‐mCherry‐WPRE (Cat# PT‐0961), rAAV‐D2‐mCherry‐WPRE (Cat# PT‐0367), rAAV‐EF1α‐DIO‐L269P‐WPRE (Cat# PT‐4107), rAAV‐EF1α‐DIO‐NLGN1‐ΔPBD‐WPRE (Cat# PT‐4105), rAAV‐EF1α‐DIO‐hChR2(H134R)‐mCherry‐WPRE (Cat# PT‐0002), rAAV‐EF1α‐DIO‐hM3D(Gq)‐mCherry‐WPRE (Cat# PT‐0042), rAAV‐EF1α‐DIO‐hM4D(Gi)‐mCherry‐WPRE (Cat# PT‐0043), rAAV‐EF1α‐DIO‐GCaMp6f‐WPRE (Cat# PT‐0106), rAAV‐EF1α‐DIO‐mCherry‐WPRE (Cat# PT‐0013), rAAV‐EF1α‐DIO‐EGFP‐WPRE (Cat# PT‐0795) viruses were purchased from BrainVTA (Wuhan, China).

### Behavioral Procedures

Prior to behavioral testing, all scheduled mice should be placed in the testing room for at least 60 min. After completing an experimental trial with a mouse, the experimental apparatus must be wiped down with 75% alcohol and allowed to air out before proceeding with the next mouse's test to reduce olfactory interference. Behavioral experiments for both control and experimental groups are conducted alternately to mitigate external factors of disturbance. Clozapine N‐oxide (CNO) (0.3 mg kg^−1^ or 1 mg kg^−1^, intraperitoneal (i.p.) injection, Sigma, Cat# C0832) was injected 30 min before the onset of behavioral tests. In intracerebral cannula injection experiments, saline or Go6983 (30 µM, 1 µL) was injected 30 min before the tests.

**Open Field Test**: The subject mice were introduced into a 50 × 50 × 50 cm apparatus and allowed to explore the open field for 10 min. The spontaneous activity of mice was captured by recording equipment. The movement parameters, such as distance, velocity and time spent in the central zone, can be calculated by Ethovision XT software.

**Spontaneous Grooming Behavior**: The subject mice were placed in a clear, empty cage measuring 29.5 × 19 × 12.5 cm and allowed to move freely for 10 min. The spontaneous activities of the mice were recorded by cameras positioned at the top, front, and sides of the cage. Grooming behavior was defined as the act of rubbing the face, body, or head with the forelimbs. The duration and frequency of grooming behavior during the 10‐minute observation period were calculated through manual analysis.

**Spontaneous Digging Behavior**: The subject mice were placed in a cage measuring 29.5 × 19 × 12.5 cm, which contained clean bedding material, and were allowed to move freely for 3 min. Their spontaneous activities were recorded by cameras positioned in three different directions. Digging behavior was defined as the act of digging or shifting bedding using either the front or hind legs. The duration and frequency of digging behavior during the 3‐minute observation period were determined through manual analysis.

**Three‐Chamber Social Test**: The three‐chamber apparatus (60 × 40 × 22 cm) is divided into three compartments: the left side, the right side, and the central chamber. Small doors at the connections between these compartments can be opened or closed to restrict the movement of the mice. The three‐chamber social interaction experiment consists of four stages, each lasting 10 min. Two age‐matched wild‐type mice, designated as Stranger 1 and Stranger 2, were selected for the experiment, with each housed separately from the subject mouse. Stage 1: The subject mouse was placed in the empty three‐chamber apparatus and allowed to freely explore for 10 min to acclimate to the experimental setup. Stage 2: Stranger 1 was placed into one side of the three‐chamber apparatus, and its movement was confined using cylindrical metal mesh barriers. The subject mouse was then allowed to freely explore for another 10 min. Stage 3: The doors connecting the central chamber were closed and the subject mouse was restricted to the central area for 10 min. Stage 4: The stranger 2 was placed into the opposite side of the three‐chamber apparatus, with both side doors open, allowing the subject mouse to freely explore any chamber for 10 min. Record videos of the mouse's activity during the four stages using cameras, and analyze the sniffing time of the subject mouse toward stranger 1, the empty metal mesh cylinder, and stranger 2 by Ethovision XT software.

### Stereotaxic Injection

Healthy 7‐ to 8‐week‐old mice were selected and administered 1.25% tribromoethanol via intraperitoneal injection. The surgical procedure and virus injection protocol were previously detailed in studies.^[^ ^53^ ^]^ The mice were carefully positioned onto a stereotaxic frame (RWD, Shenzhen, Cat# D01476‐002). A midline scalp incision was then performed, followed by the creation of a skull aperture using a 0.6 mm diameter drill bit. The viruses were injected into various brain regions, including the dorsal striatum, ventral striatum, mPFC, OFC, PVH, CeA (dorsal striatum: AP: 0.5 mm, DV: ‐3.10 mm, ML: ±2.05 mm relative to bregma; ventral striatum: AP: 1.20 mm, DV: ‐4.20 mm, ML: ±1.35 mm relative to bregma; mPFC: AP: 2.30 mm, DV: ‐2.0 mm, ML: ±0.3 mm relative to bregma; OFC: AP: 2.6 mm, DV: ‐2.2 mm, ML: ±0.5 mm, ±1.5 mm relative to bregma; PVH: AP: ‐0.5 mm, DV: ‐5.0 mm, ML: ±0.25 mm relative to bregma; CeA: AP: ‐1.5 mm, DV: ‐5.0 mm, ML: ±2.0 mm relative to bregma. The viruses were administered bilaterally at a rate of 0.05 µL min^−1^ using a 10 µL microliter syringe and micro‐injection pump (KD Scientific, Cat# 78–8130), with a total volume of 0.3 µL per side. After the injection, the internal cannula was left in place for 10 min to facilitate diffusion. Subsequently, the mice were placed on a heating pad to ensure full recovery. Subsequent testing on the mice injected with the virus should be conducted for 2 to 6 weeks post‐injection to assess virus expression and effects.

### Optic Fiber Implantation and Optogenetic Experiments

For optic fiber implantation, mice were anesthetized with 1.25% tribromoethanol administered via intraperitoneal injection. After 2 to 3 weeks of viral expression, the skull was re‐exposed, and optical fibers (200 µm diameter, 0.37 NA, 3.0 mm length, fixed on 1.25 mm outer diameter ceramic ferrules) (Inper, Hangzhou, China) were implanted at the designated coordinates (AP: 0.5 mm, ML: ±2.05 mm, DV: ‐3.05 to ‐3.00 mm), adjusted to be 0.05 to 0.1 mm higher than the virus injection site. Behavioral experiments were conducted one week after recovery. For the D2‐MSN activation experiments, blue light (473 nm, 4 mW, pulse width of 10 ms, 10 Hz/20 Hz/30 Hz/40 Hz) from a laser stimulator (Aurora‐220, Newdoon, Hangzhou, China) was delivered bilaterally into the dorsal striatum via a mono‐optogenetic fiber (Inper, Hangzhou, China).

### Chemogenetic Experiments

For the experiments involving D2‐MSN activation (hM3DG(q)), CNO was administered intraperitoneally at doses of 0.3 mg kg^−1^ or 1 mg kg^−1^, 30 min prior to the start of the experiment. In the D2‐MSN inhibition experiment, *Nlgn1* KO mice were injected with a chemically inhibited virus (hM4DG(i)) into the dorsal striatum. After a two‐week interval, half of the mice received saline injections as a control, while the other half received CNO injections as the experimental group. One week later, the original experimental group was given saline injections to serve as the control group, while the initial control group received CNO to function as the experimental group. The same cohort of mice participated in both experiments. The site of virus injection was confirmed at the conclusion of the experiment.

### Cannula Implantation

For cannula implantation for drug delivery, 2‐month‐old WT mice or *Nlgn1* KO mice were anesthetized with 1.25% tribromoethanol through intraperitoneal injection. The cannulas (RWD, Shenzhen, Cat# 62003) were bilaterally implanted into the dorsal striatum (AP: 0.5 mm, DV: ‐3.05 mm, ML: ±2.05 mm relative to bregma), with one screw placed on each side of the skull to form a stable structure with the cannulas. Dental cement was applied over the skull to secure the guide cannulas, and caps (RWD, Shenzhen, Cat# 62102) were used to protect the guide cannulas. Mice were placed on a heating pad for a full recovery. The experiments were conducted 1 week after cannula implantation.

### Intracerebral Cannula Drug Injections

For intracerebral cannula drug injections, WT mice or *Nlgn1* KO mice were anesthetized with 4% isoflurane and maintained under 1% isoflurane anesthesia before being positioned on a stereotaxic frame. Each mouse received bilateral injections of 1 µL of drug, administered at a rate of 0.3 microliters per minute. The specific drug concentrations were as follows: 30 µM Go6983, 100 µM NBQX, 250 µM AP5, 50 µM H89, and 10 µM AG126. Fiber optic drug delivery tubes (Fiber diameter 200 µm, NA value 0.37, ceramic connector OD 1.25 mm) (KedouBC, Suzhou) were used for the experiment of optical fiber recording and drug combination. These tubes can be replaced with cartridge needles for fiber‐optic recording after drug injection completed. After the injections, the mice were placed in a housing cage for 30 min prior to behavioral tests.

### Immunohistochemistry

Following the brain tissue sectioning protocol described in the previous article,^[^ ^54^, ^55^ ^]^ mice were anesthetized with 1.25% tribromoethanol. They were then perfused with 30 mL of phosphate buffer (PBS, Cat# 311‐010‐CL, Wisent), followed by perfusion with 30 mL of 4% paraformaldehyde (PFA, Cat# A500684, Sangon Biotech) in PBS. Subsequently, the brain tissue was dissected, fixed in PFA for 20 h, and then transferred to 30% sucrose solution until completely saturated. The brain was embedded in OCT (Cat# 4583, Sakura), kept at 4 °C for 20 min, and then rapidly frozen with liquid nitrogen. Finally, the brain was stored at ‐20 °C (Leica CM1950) and sectioned into 30 µm coronal or sagittal slices. The sections were washed three times with PBS and permeabilised with PBS containing 0.3% Triton X‐100 for 1 h. The samples were then incubated with PBS containing 10% FBS for 2 h at room temperature and incubated with primary antibody in PBS overnight at 4 °C. The sections were rinsed three times with PBS and incubated with the appropriate secondary antibody in PBS at 37 °C for 2 h. The sections were washed three times with PBS and carefully placed onto glass slides, followed by two washes. After air‐drying, they were mounted using a mounting medium containing DAPI. The coverslips were delicately positioned over the slides to seal them. The images were obtained by BioTek imaging reader (BioTek cytation5). Antifade Mounting Medium with DAPI was from Beyotime (Cat# P0131, Beyotime).

### Western Blot Analysis

Referring to the previous experimental method,^[^ ^55^ ^]^ mice were euthanized by acute decapitation, and their brain tissue was promptly dissected to isolate the striatal structures. In the case of mice injected with viruses, brain regions labeled with fluorescence were specifically dissected. The dissected tissue was then placed into a protein lysis buffer containing 2.5 mM sodium pyrophosphate, 150 mM NaCl, 1 mM EDTA, 1 mM EGTA, 0.5% NP‐40, 20 mM Tris (pH 7.5), 1 mM β‐glycerophosphate, 1 mM Na_3_VO_4_, 20 mM NaF, and 1% protease inhibitor cocktail and phosphatase inhibitor (Cat# A32961, Thermo). Following homogenization on ice for 40–60 min, the lysate was centrifuged at 12 000 rpm for 10 min at 4 °C. The supernatant was collected and mixed with 5×SDS‐loading buffer (10% SDS, 0.5% bromophenol blue, 50% glycerol, 250mM Tris‐HCl, 5% β‐mercaptoethanol, pH 7.4) for electrophoresis on an SDS‐PAGE gel. Subsequently, the gel was transferred onto a PVDF filter. The filter was then blocked with 5% skim milk in TBST (9% NaCl, 20 mM Tris, 1% Tween‐20, pH 7.6) and incubated overnight at 4 °C with appropriate primary antibodies diluted in TBST. After washing and incubation with a secondary antibody, the membrane was washed again and subjected to an enhanced chemiluminescence assay (Cat# 34577, Thermo). Protein bands were analyzed using AlphaEaseFC software following the manufacturer's instructions. The total protein loading was standardized by normalizing each tested protein with the immunoreactivity of GAPDH on the same filter.

### Slice Electrophysiology

The detailed procedures for acute brain slice recordings were described previously.^[^ ^55^ ^]^ All recordings were conducted at the dorsal striatum of 2–5‐month‐old mice. In brief, the mouse brains were quickly removed, and sagittal brain slices (300 µm) were prepared (Leica, VT1000S) in ice‐cold artificial cerebrospinal fluid (ACSF) containing (in mM): 120 NaCl, 3.0 KCl, 1.2 MgSO4, 1.0 NaH2PO4, 26 NaHCO3, 2.0 CaCl2, 11 D‐glucose saturated with 95% O2/5% CO2. The slices were moved to 95% O2/5% CO2 saturated ACSF at 28 °C for at least 2 h before being transferred to a chamber perfused with oxygen and carbon dioxide saturated ACSF. The mCherry‐positive D2‐MSNs were selected for recording based on the expression of the fluorescent marker using an upright Olympus BX51WI microscope equipped with the appropriate filters (Olympus, Japan) and an LED light engine (CoolLED‐pE100, UK). The drugs used for recordings included: CNQX (10 µM, Cat# N183, Sigma), NBQX (20 µM, Cat# N171, Sigma), Picrotoxin (PTX) (100 µM, Cat# R284556, Sigma), AP5 (50 µM, Cat# 0106, Tocris) and Go6983 (20 µM). For current injection recording, D2‐MSNs were recorded under current clamp model with glass pipettes (3–7 MΩ) filled with the intracellular solution containing (in mM) 130.0 C_6_H_11_KO_7_, 5.0 NaCl, 1.0 MgCl_2_, 0.05 EGTA, 10.0 HEPES, 3.0 Mg‐ATP, 0.3 Na_3_GTP (pH 7.25) (280–300 mOsm), and injected current steps (from 0 to +250 pA, 50 pA step). For confirmation of ChR2 mediated optical activation, D2‐MSNs were clamped at current clamp model, and different frequencies of light (1 s, 473 nm, 4 mW) were delivered from a laser stimulator (Aurora‐220, Newdoon, Hangzhou, China) to evoke potential alteration. For voltage‐dependet potassium current recording, the intracellular solution was containing (in mM): 140.0 C_6_H_11_KO_7_, 10.0 KCl, 10.0 HEPES, 0.1 EGTA, 2.0 MgCl_2_, 2.0 Mg‐ATP and 5.0 QX‐314 (pH 7.25) (280–300 mOsm). Neurons were held at ‐30 mV for 500 ms to inactivate A‐type potassium channels, and then clamped from ‐80 mV to +40 mV (10 mV per step, lasting 5 s). The activation curves of voltage‐dependent potassium currents were measured by assessing the outward currents.

For voltage‐dependet sodium current recording, the intracellular solution was containing (in mM): 145.0 CsCl, 2.0 TEA‐Cl, 0.2 EGTA, 10.0 HEPES, 2.0 MgCl_2_ and 2.0 Mg‐ATP (pH 7.25) (280–300 mOsm), with the extracellular ACSF containing 3.0 mM 4‐AP and 0.1 mM CdCl_2_. We recorded Na^+^ currents elicited by consecutive depolarizing voltage steps from ‐70 mV to +40 mV (10 mV per step, lasting 30 ms) following a prepulse of ‐100 mV. The activation curves were generated based on the peak currents at each step and the conductance of the neuron. For mEPSC recording, cells were clamped at ‐70 mV under voltage clamp model, and incubated in the ACSF containing 100 µM picrotoxin. For mIPSC recording, D2‐MSNs were clamped at 0 mV under voltage clamp model, and incubated in the ACSF containing 10 µM CNQX and 50 µM AP5. The intracellular solutions used for mEPSC and mIPSC recording were containing (in mM): 130.0 CsMeSO_4_, 5.0 NaCl, 1.0 MgCl_2_, 0.05 EGTA, 10.0 HEPES, 3.0 Mg‐ATP, 0.3 Na_3_GTP and 5.0 QX‐314 (pH 7.25) (280–300 mOsm). During the recordings, cell series resistance was monitored throughout experiments by applying a (‐3 mV) step at the end of each sweep and the experiment was excluded from analysis if the resistance changed by more than 20%. All data acquisition and analysis were done using pCLAMP 10.2 (Molecular Devices) and Mini Analysis software (Synaptosoft). In all electrophysiological experiments, n represents the number of cells and normally three or four cells per animal were used.

### Fiber Photometry Recording In Vivo

Following the previously described method,^[^ ^53^, ^54^ ^]^ the calcium indicator virus was injected into the dorsal striatum, with fibers implanted two weeks later. After a recovery period of one week, Ca^2+^ signals from D2‐MSN neurons were recorded by fiber photometry system. To examine the relationship between spontaneous stereotypic behavior and Ca^2+^ signaling in dorsal D2‐MSNs, we placed the subject mice in either a clean cage or a cage with bedding and let them move freely for ten minutes. Animal behaviors and calcium signals were recorded simultaneously. Analog voltage signals were digitized at a rate of 50 Hz and recorded by fiber photometry system (QAXK‐FPS‐MC‐LED, ThinkerTech, Nanjing, China). Subsequently, the digitized data were analyzed further using customized software developed in MatLab (Natick, USA). For fiber photometry Ca^2+^ imaging data analysis, the dF/F_0_ value was calculated with the algorithm: dF/F_0_ = (F‐F_0_)/F_0_. For each recording, F_0_ was the mode of the entire fluorescence recording. For accumulated calcium event (ACE) analysis, if the peak amplitude of an event exceeded twice the average cumulative fluorescence intensity, we defined it as an ACE event. After the experiment, the virus expression and fiber position were examined for each mouse.

### Single Nucleus Isolation and RNA Sequencing

The nucleus was isolated using the Shbio Nuclei Isolation Kit (SHBIO, #52009‐10, China). Briefly, fresh striatal tissues of WT and *Nlgn1* KO mice were quickly isolated and added to the lysis buffer. The tissues were then homogenized to a liquid state using a tissue homogenizer. The resulting tissue lysates were passed through a 40 µm cell sieve to remove impurities and transferred to a new 2 mL EP tube. The samples were centrifuged at 500 g at 4 °C for 5 min to obtain the precipitate. Next, PB1, PB2, and PB3 solutions were added to the precipitate, with the nuclei located at the interface of the PB2 and PB3 solutions. Finally, the nuclei were resuspended by gently pipetting into 50 µL of NB solution. The nuclei were counted using a cell counter (Thermo Fisher, USA).

Using a Chromium Single Cell 3′ Library and Gel Bead Kit v3 (10X Genomics, USA), nuclei were immediately loaded onto a Chromium Single Cell Processor (10X Genomics, USA) for the barcoding of RNA from individual nuclei. Sequencing libraries were constructed according to the manufacturer's instructions (10X Genomics, USA) and subsequently sequenced on a NovaSeq 6000 sequencing system (Illumina, USA). Reads were processed using the Cell Ranger 2.1.0 pipeline with default and recommended parameters. FASTQ files generated from Illumina sequencing output were aligned to the mouse genome, version GRCm38, using the STAR algorithm. Next, Gene‐Barcode matrices were generated for each individual sample by counting unique molecular identifiers (UMIs) and filtering out non‐cell‐associated barcodes. Finally, the gene‐barcode matrix containing the barcoded cells and gene expression counts was created. The outputs were then imported into the Seurat (v2.3.0) R toolkit for quality control and downstream analysis of our single‐cell RNA‐seq data. Nuclei with fewer than 200 or more than 6000 detected genes were excluded. Normalization of the data (using the Normalize Data function in the Seurat package) was performed to extract a subset of variable genes. Variable genes were identified while controlling for the strong relationship between variability and average expression. Principal component analysis (PCA) was conducted to reduce the data to the top 30 PCA components after scaling the data.

### Statistical Analysis

All data were presented as mean ± standard error of the mean (mean ± s.e.m.) and statistically analyzed using two‐tailed unpaired *t*‐test, two‐tailed paired *t*‐test, one‐way analysis of variance (one‐way ANOVA), two‐way analysis of variance (two‐way ANOVA), or repeated‐measurements (RM), as appropriate, followed by Fisher's LSD post hoc test. A p‐value less than 0.05 was considered significant (*p < 0.05, **p < 0.01, ***p < 0.001). Data analysis was conducted by GraphPad 9 software. Graph drawings were performed by GraphPad 9 and Adobe Illustrator CC 2020. The specific statistical methods and values were displayed in the figure legends and Tables S1 (Main figures) and S2 (Extended figures) (Supporting Information).

## Conflict of Interest

The authors declare no conflict of interest.

## Author Contributions

D.L. and A.L. contributed equally to this work. A.L. and W.X. performed the conceptualization. A.L., D.L., Z.J., and J.H. performed the methodology. D.L., A.L., M.M., M.W., X.L., K.C., and R.L. performed the investigation. A.L., W.X. performed the funding acquisition. A.L. and W.X. performed the supervision. A.L., D.L., and W.X. performed writing, review & editing.

## Supporting information



Supporting Information

Supplemental Table 1

Supplemental Table 2

Supplemental Table 3

Supplemental Table 4

## Data Availability

The data that support the findings of this study are available on request from the corresponding author. The data are not publicly available due to privacy or ethical restrictions.
