# Supplementary material for: Neuroligin 1 Regulates Autistic‐Like Repetitive Behavior through Modulating the Activity of Striatal D2 Receptor‐Expressing Medium Spiny Neurons
Source: Adv Sci (Weinh). 2024 Dec 11;12(5):2410728. doi: 10.1002/advs.202410728 (PMC11792054; doi:10.1002/advs.202410728)
Supplement: Supplementary file 1 — Supporting Information [file ADVS-12-2410728-s005.docx]

**Supplementary materials**

**Figures and Legends**

**
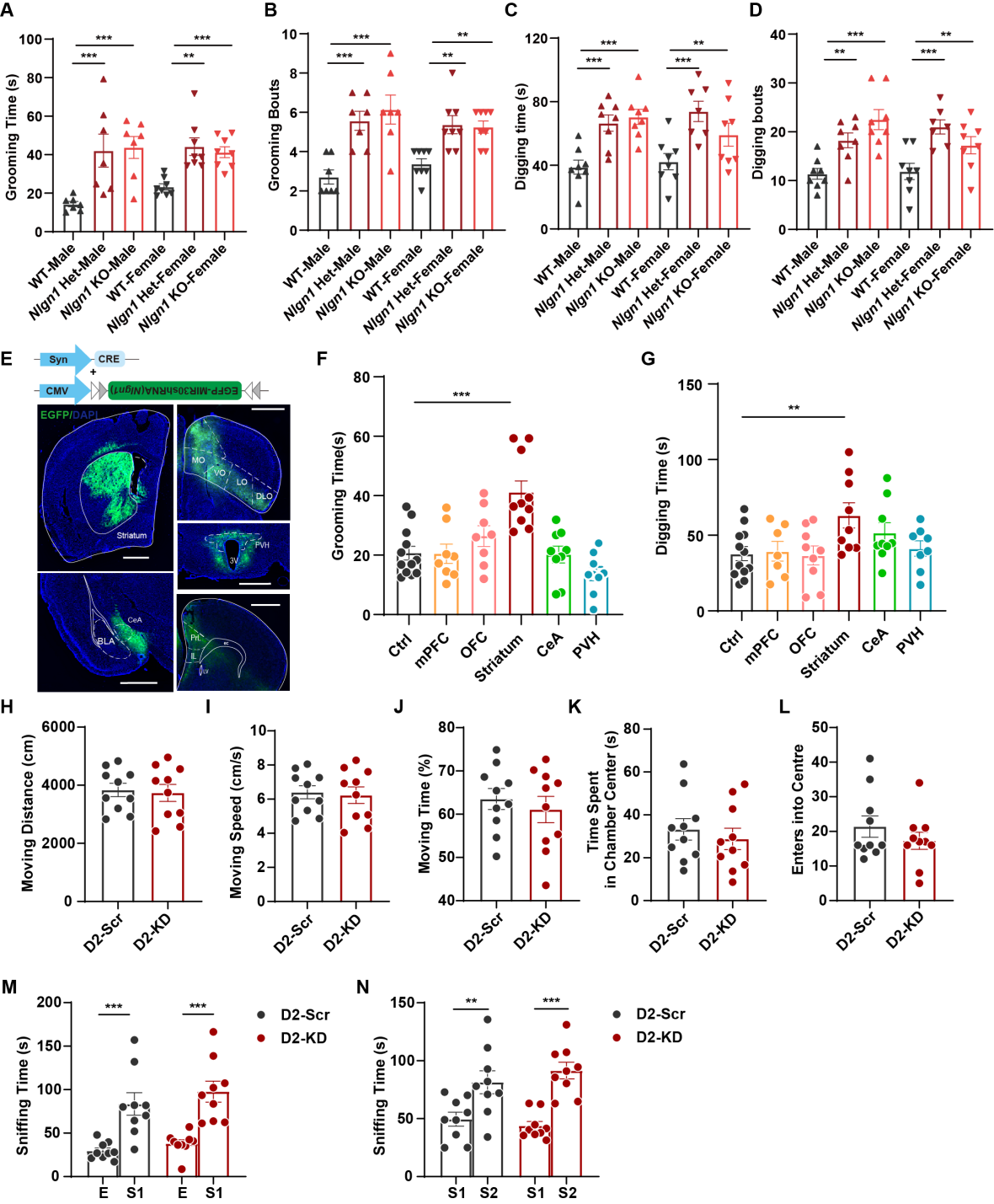
**

**Figure S1**

A-B. Statistical graphs of self-grooming time (A) and bouts (B) in WT-Male, *Nlgn1* Het-Male, *Nlgn1* KO-Male, WT-Female, *Nlgn1* Het-Female and *Nlgn1* KO-Female mice.

C-D. Statistical graphs of digging time (C) and bouts (D) in WT-Male, T-Male, *Nlgn1* Het-Male, *Nlgn1* KO-Male, WT-Female, *Nlgn1* Het-Female and *Nlgn1* KO-Female mice.

E. Illustration and sample images of Syn-driven scramble (Scr) or *Nlgn1* knockdown (KD) virus in multiple brain regions. Scale bar: 1 mm.

F-G. Statistical graphs of self-grooming time (F) and digging time (G) in *Nlgn1* KD virus expressed multi-groups.

H-L. Statistical graphs of moving distance (H), moving speed (I), moving time (J), time spent in chamber center (K) and enters into center (L) of D2-driven Scr and *Nlgn1* KD viruses expressed mice.

M. Statistical graphs of sniffing time of D2-driven Scr snd *Nlgn1* KD viruses expressed mice to empty cage and S1 stranger mouse.

N. Statistical graphs of sniffing time of D2-driven Scr snd *Nlgn1* KD viruses expressed mice to S1 and S2 stranger mice.

Data represent mean ± SEM; Two-way ANOVA with Fisher's LSD post hoc test for panel A-D and M-N, one-way ANOVA test with LSD post hoc multiple comparisons for panel F and G, two-tailed unpaired t-test for panel H-L. For all the panels, dots represent individual mice. *p< 0.05; **p< 0.01, ***p<0.001. Also see Table S2.


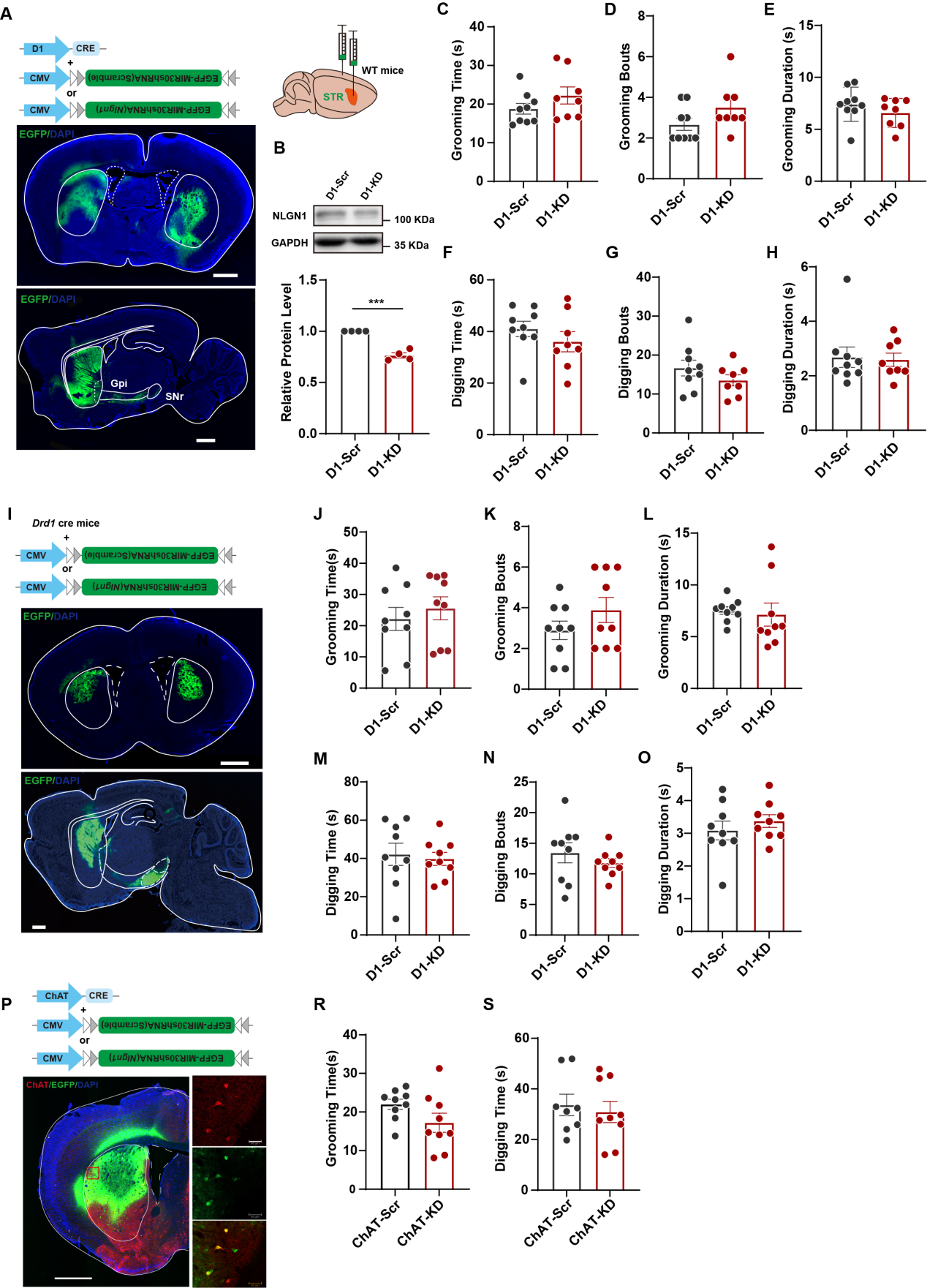


**Figure S2**

A. Illustration and sample images of D1-driven Scr or *Nlgn1* KD in striatum. Scale bar: 1mm.

B. Representative images (upper) and statistical graph (lower) of NLGN1 protein expression in striatum tissues from mice depicted in panel (A).

C-E. Statistical graphs of self-grooming time (C), bouts (D) and average grooming duration per bout (E) in the mice depicted in panel (A).

F-H. Statistical graphs of digging time (F), bouts (G) and average digging duration per bout (H) in the mice depicted in panel (A).

I. Illustration and sample images of *Nlgn1* KD expression in dorsal striatum of *Drd1*-Cre mice. Scale bar: 1mm.

J-L. Statistical graphs of self-grooming time (J), bouts (K) and average grooming duration per bout (L) in *Drd1*-Cre mice depicted in panel (I).

M-O. Statistical graphs of digging time (M), bouts (N) and average digging duration per bout (O) in *Drd1*-Cre mice depicted in panel (I).

P. Illustration and sample images of ChAT-driven Scr or *Nlgn1* KD in striatum, the reliability of ChAT-Cre virus was verified by ChAT antibody immunostaining. Scale bar: left,1 mm; right, 50μm.

R-S. Statistical graphs of self-grooming time (R) and digging time (S) in the mice depicted in panel (P).

Data represent mean ± SEM; Two-tailed unpaired t-test for panel B-H, J-O and R-S. For all the panels, dots represent individual mice. Also see Table S2.


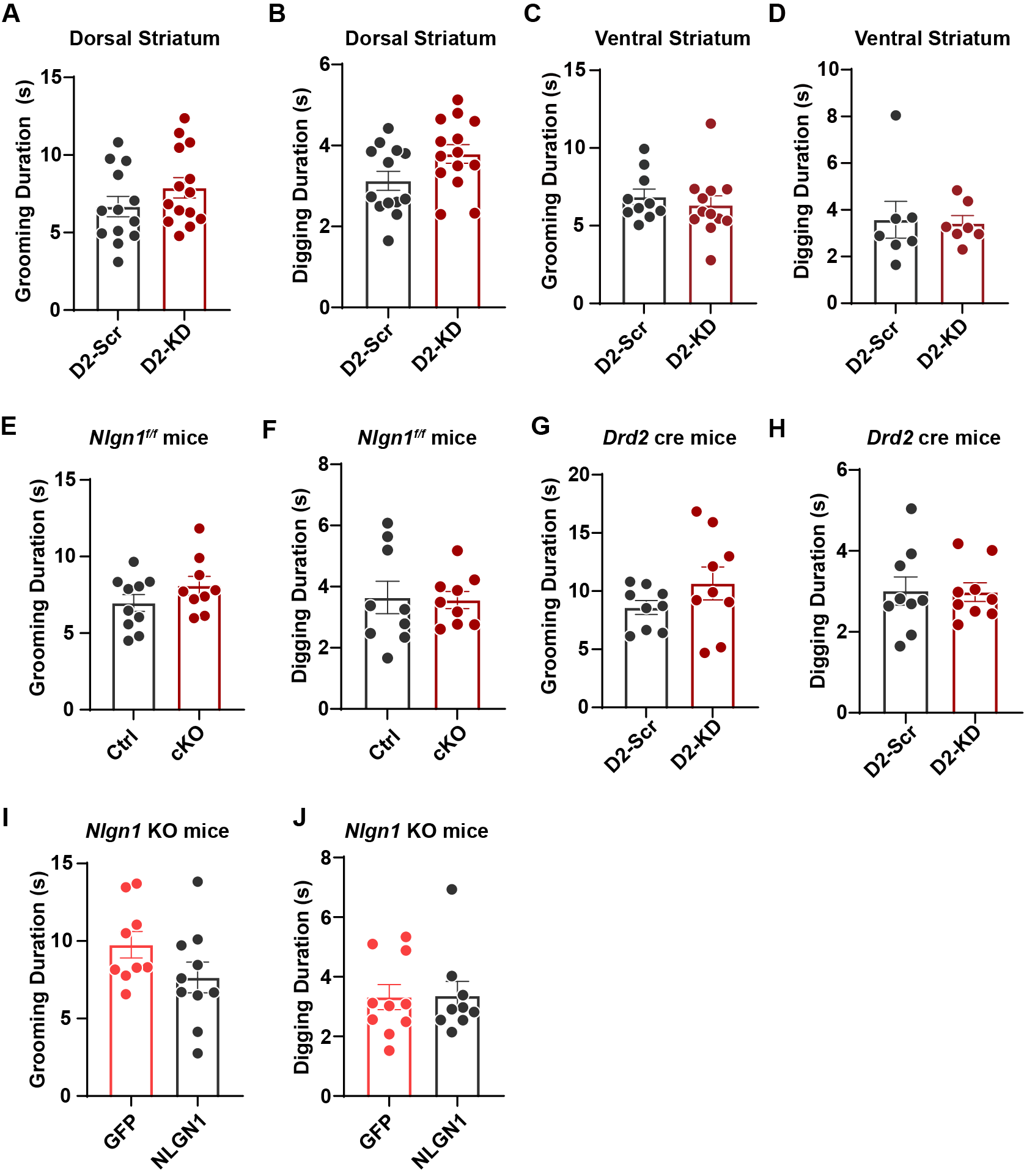


**Figure S3**

A-B. Statistical graphs of average grooming (A) and digging (B) duration per bout in dorsal striatal D2-driven Scr and *Nlgn1* KD viruses expressed mice.

C-D. Statistical graphs of average grooming (C) and digging (D) duration per bout in ventral striatal D2-driven Scr and *Nlgn1* KD viruses expressed mice.

E-F. Statistical graphs of average grooming (E) and digging (F) duration per bout in *Nlgn1^f/f^* mice without (Ctrl) or with Cre (cKO) expression in dorsal D2-MSNs.

G-H. Statistical graphs of average grooming (G) and digging (H) duration per bout in dorsal striatal scramble and *Nlgn1* KD viruses expressed *Drd2*-Cre mice.

I-J. Statistical graphs of average grooming (I) and digging (J) duration per bout in dorsal striatal GFP and HA-NLGN1 viruses expressed *Nlgn1* KO mice.

Data represent mean ± SEM; Two-tailed unpaired t-test for all panels. For all the panels, dots represent individual mice. Also see Table S2.


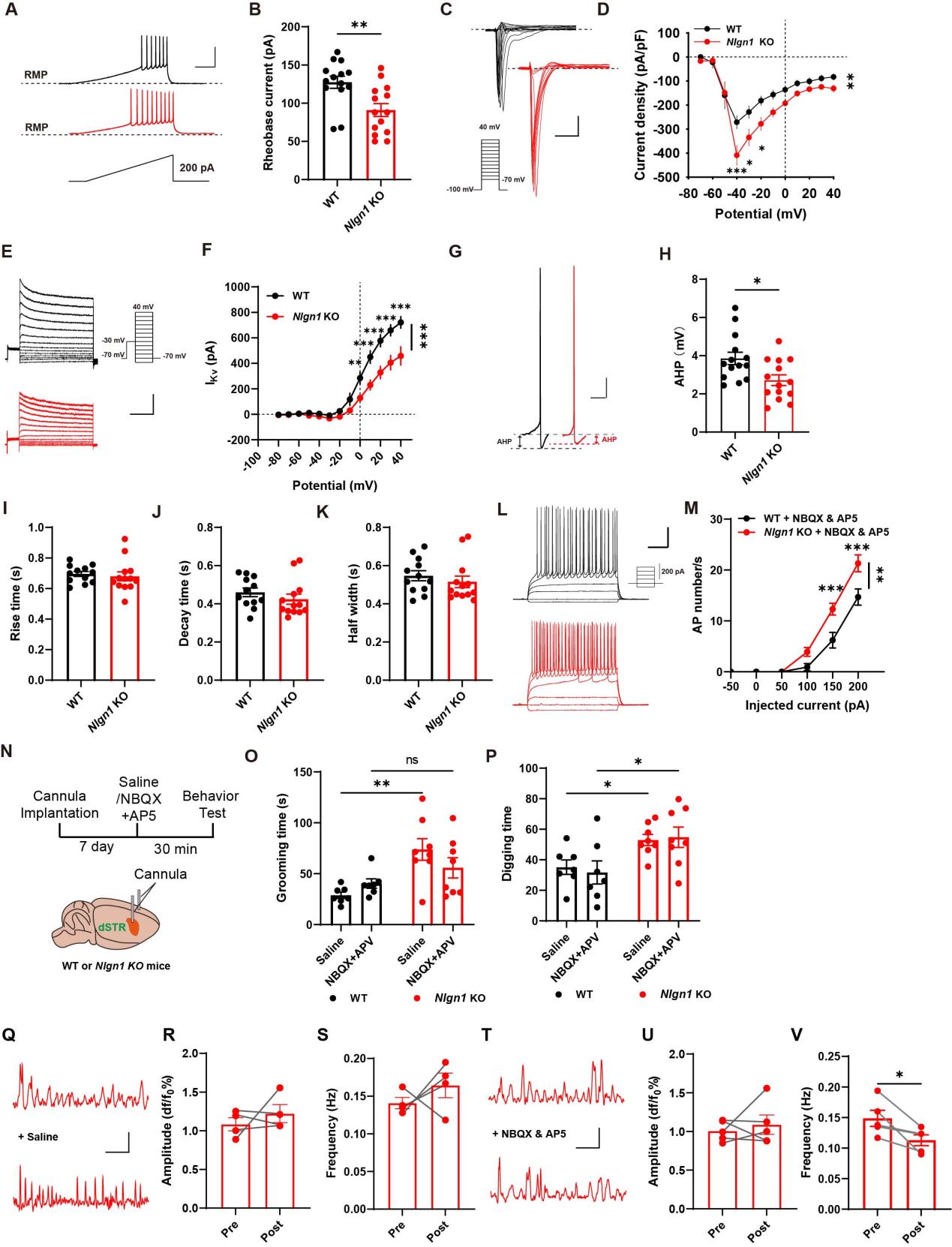


**Figure S4**

1. Illustration and sample traces of the electrophysiological responses during 200 pA ramp current injection in WT and *Nlgn1* KO D2-MSN. Scale bar: 0.2 s / 40 mV.
2. Statistical graph of the rheobase current in WT and *Nlgn1* KO D2-MSNs.
3. Illustration and sample traces of the voltage-step protocol (below) induced voltage-dependent sodium currents in WT and *Nlgn1* KO D2-MSNs. Scale bar: 5 ms / 1 nA.
4. The current–voltage relationship (I–V) curve of sodium current density (pA/pF) in in WT and *Nlgn1* KO D2-MSNs.
5. Illustration and sample traces of the voltage-step protocol (right) induced voltage-dependent potassium currents in WT and *Nlgn1* KO D2-MSNs. Scale bar: 1.5 s / 0.5 nA.
6. The activation I–V curve of voltage-gated potassium current in WT and *Nlgn1* KO D2-MSNs.
7. Illustration and sample traces of the AHP of APs in WT and *Nlgn1* KO D2-MSNs. Scale bar: 0.1 s / 8 mV.
8. Statistical graph of the AHPs in WT and *Nlgn1* KO D2-MSNs.
9. K. Statistical graphs of average rise time (I), decay time (J) and half width (K) of the Ca^2+^ events of WT and *Nlgn1* KO D2-MSNs.
10. Current injection intensity illustration and sample traces of injecting current evoked action potentials in NBQX + AP5 treated WT and *Nlgn1* KO D2-MSNs. Scale bar: 0.2 s / 25 mV.

M. Statistical curves of injecting currents evoked action potential numbers in NBQX + AP5 treated WT and *Nlgn1* KO D2-MSNs.

N. Experiment strategy of cannula implantation and NBQX + AP5 injection in *Nlgn1* KO or WT striatum.

O-P. Statistical graphs of grooming (O) and digging (P) time in the dorsal striatal region of saline and NBQX + AP5 injected WT and *Nlgn1* KO mice.

Q. Sample traces of Ca^2+^ signals of *Nlgn1* KO dorsal striatal D2-MSNs before and after saline injection. Scale bar: 10 s / 1% df/f_0_.

R-S. Statistical graphs of the amplitude (R) and frequency (S) of Ca^2+^ events of *Nlgn1* KO dorsal striatal D2-MSNs before and after saline injection.

T. Sample traces of Ca^2+^ signals of *Nlgn1* KO dorsal striatal D2-MSNs before and after NBQX + AP5 injection. Scale bar: 10 s / 1% df/f_0_.

U-V. Statistical graphs of the amplitude (U) and frequency (V) of Ca^2+^ events of *Nlgn1* KO dorsal striatal D2-MSNs before and after NBQX + AP5 injection.

Data represent mean ± SEM; Two-tailed unpaired t-test for panel B, I-K, repeated two-way ANOVA with Fisher's LSD post hoc test for panel D, F and M, two-way ANOVA with Fisher's LSD post hoc test for panel O and P, two-tailed paired t-test for panel R-S and U-V. For panel B and H, dots represent individual neurons, for panel I-K, dots represent individual Ca^2+^ traces, for panel O, P, R, S, U and V, dots represent individual mice. *p < 0.05, **p < 0.01, ***p < 0.001. Also see Table S2.


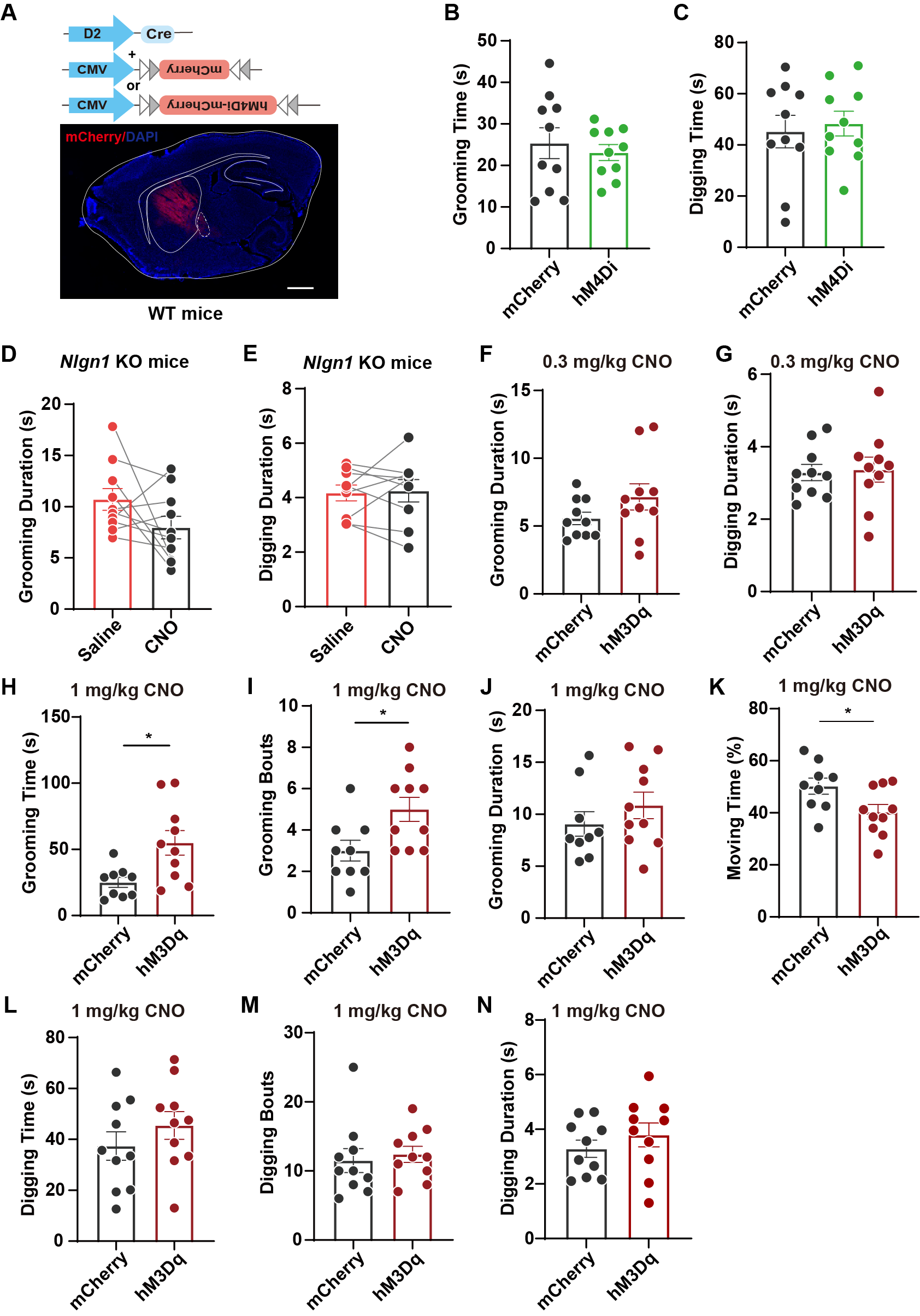


**Figure S5**

1. Illustration and sample image of D2-driven hM4d(Gi)-mCherry expression in dorsal striatum of WT mice. Scale bar: 1 mm.

B-C. Statistical graphs of self-grooming time (B) and digging time (C) in the mice depicted in panel (A).

D-E. Statistical graphs of grooming (D) and digging (E) duration per bout in saline and CNO injected dorsal striatal D2-driven hM4d(Gi) expressed *Nlgn1* KO mice.

F-G. Statistical graphs of grooming (F) and digging (G) duration per bout in 0.3 mg/kg CNO treated dorsal striatal D2-MSN mCherry and hM3d(Gq) expressed WT mice.

H-J. Statistical graphs of self-grooming time (H), bouts (I), and grooming duration per bout (J) in 1 mg/kg CNO treated dorsal striatal D2-MSN mCherry and hM3d(Gq) expressed WT mice.

K. Statistical graph of moving time percentage in 1 mg/kg CNO treated dorsal striatal D2-MSN mCherry and hM3d(Gq) expressed WT mice.

L-N. Statistical graphs of digging time (L), bouts (M), and digging duration per bout (N) in 1 mg/kg CNO treated dorsal striatal D2-MSN mCherry and hM3d(Gq) expressed WT mice.

Data represent mean ± SEM; Two-tailed unpaired t-test for panel B-C and F-N, two-tailed paired t-test for panel D and E. For all the panels, dots represent individual mice. *p< 0.05. Also see Table S2.


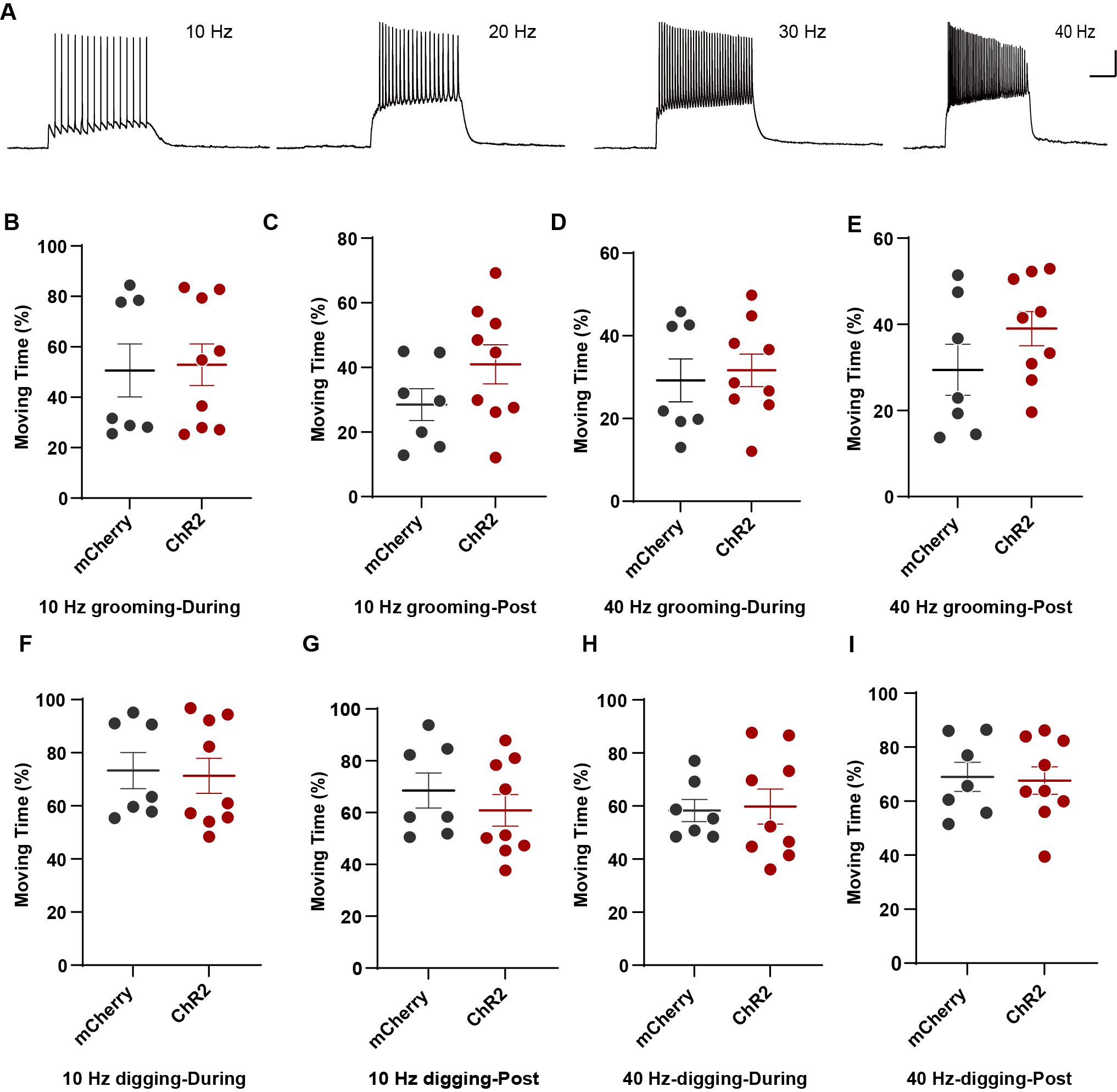


**Figure S6**

A. Sample traces of optogenetic light stimulation at different frequencies evoked action potentials in ChR2 expressed D2-MSN in WT slices. Scale bar: 0.25 s / 15 mV.

B-C. Statistical graphs of moving time percentage of D2-MSN ChR2 expressing mice in cages without bedding during (B) and post (C) 10 Hz stimulation.

D-E. Statistical graphs of moving time percentage of D2-MSN ChR2 expressing mice in cages without bedding during (D) and post (E) 40 Hz stimulation.

F-G. Statistical graphs of moving time percentage of D2-MSN ChR2 expressing mice in cages with bedding during (F) and post (G) 10 Hz stimulation.

H-I. Statistical graphs of moving time percentage of D2-MSN ChR2 expressing mice in cages with bedding during (H) and post (I) 40 Hz stimulation.

Data represent mean ± SEM; Two-tailed t-test for panel B-I and dots represent individual mice. Also see Table S2.


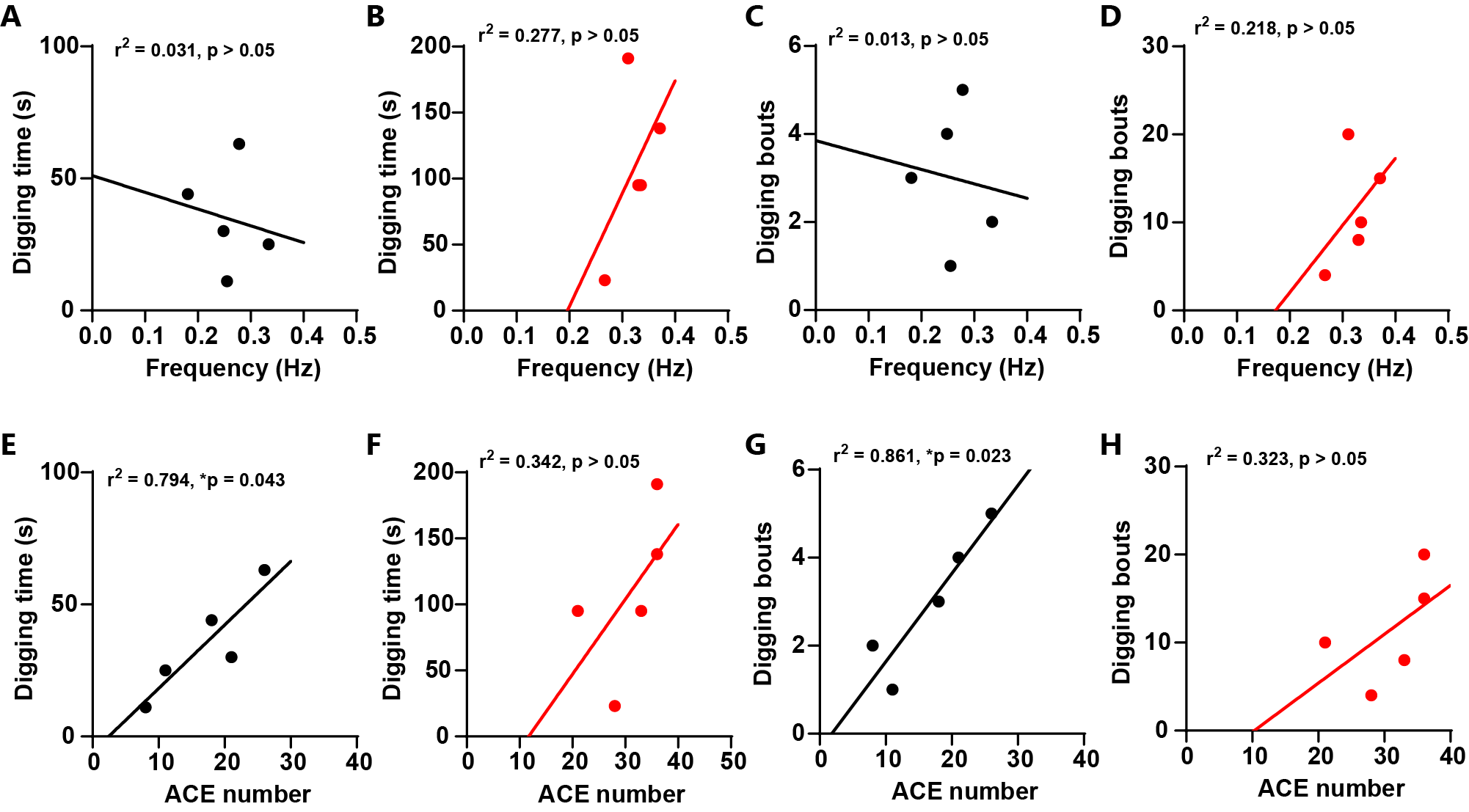


**Figure S7**

1. B. Correlation matrix analysis of digging time and Ca^2+^ event frequency in WT (A) and *Nlgn1* KO (B) mice.

C-D. Correlation matrix analysis of digging bouts and Ca^2+^ event frequency in WT (C) and *Nlgn1* KO (D) mice.

E-F. Correlation matrix analysis of digging time and ACE number in WT (E) and *Nlgn1* KO (F) mice.

G-H. Correlation matrix analysis of digging bouts and ACE number in WT (G) and *Nlgn1* KO (H) mice.

Correlation matrix analysis was performed for panel A-H. For all the panels, dots represent individual mice. *p<0.05. Also see Table S2.


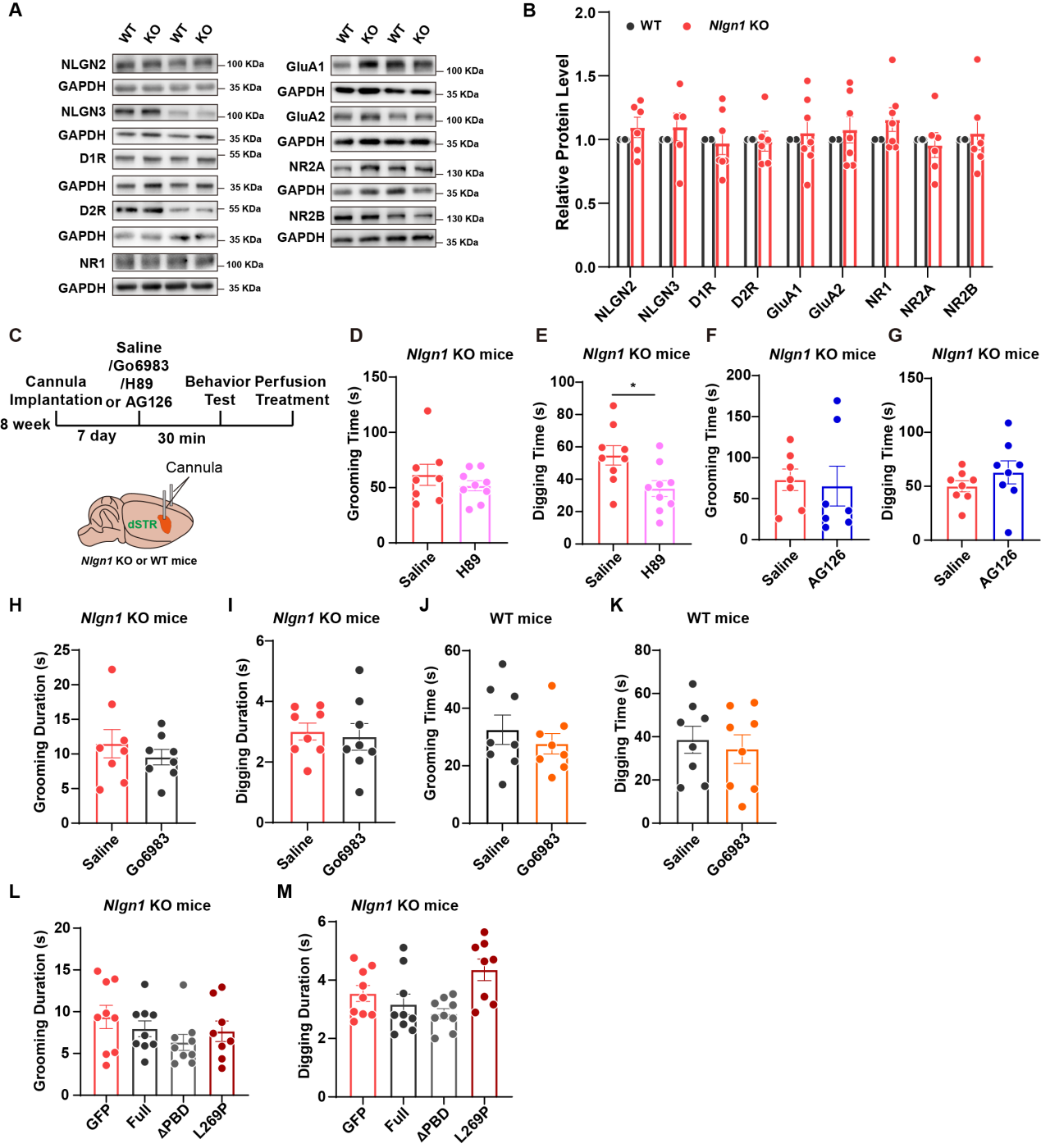


**Figure S8**

A-B. Representative western blot images (A) and quantification (B) of synaptic protein changes in the striatal tissues of WT and *Nlgn1* KO mice.

C. Experiment strategy of cannula implantation and drug injection in *Nlgn1* KO or WT dorsal striatum.

D-E. Statistical graphs of self-grooming time (D) and digging time (E) in saline and H89 injected *Nlgn1* KO mice.

F-G. Statistical graphs of self-grooming time (F) and digging time (G) in saline and AG126 injected *Nlgn1* KO mice.

H-I. Statistical graphs of average grooming (H) and digging duration per bout (I) in saline and Go6983 injected *Nlgn1* KO mice.

J-K. Statistical graphs of self-grooming time (J) and digging time (K) in saline and Go6983 injected WT mice.

L-M. Statistical graphs of average grooming (L) and digging duration per bout (M) in striatal D2-driven GFP, NLGN1-full,NLGN1-ΔPBD and NLGN1-L269P viruses expressed *Nlgn1* KO mice.

Data represent mean ± SEM; Two-tailed unpaired t-test for panel B and D-K, one-way ANOVA test with LSD post hoc multiple comparisons for panel L and M. For all panels, dots represent individual mice. Also see Table S2.


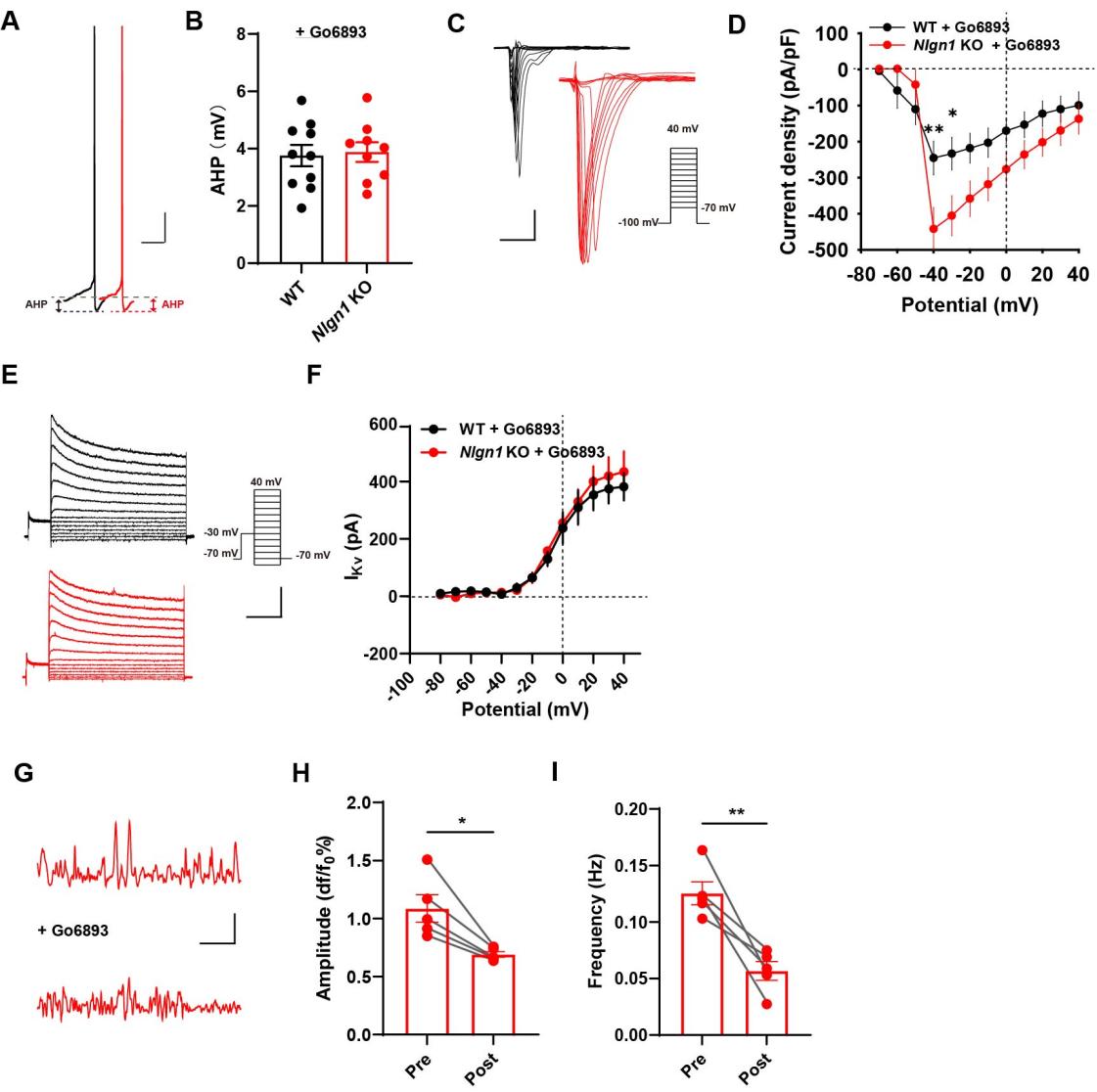


**Figure S9**

1. Illustration and sample traces of the AHP of APs in Go6893 treated WT and *Nlgn1* KO D2-MSNs. Scale bar: 0.1 s / 8 mV.
2. Statistical graph of the AHPs in Go6893 treated WT and *Nlgn1* KO D2-MSNs.
3. Illustration and sample traces of the voltage-step protocol (below) induced voltage-dependent sodium currents in Go6893 treated WT and *Nlgn1* KO D2-MSNs. Scale bar: 5 ms / 1 nA.
4. The I-V curve of sodium current density (pA/pF) in in Go6893 treated WT and *Nlgn1* KO D2-MSNs.
5. Illustration and sample traces of the voltage-step protocol (right) induced voltage-dependent potassium currents in Go6893 treated WT and *Nlgn1* KO D2-MSNs. Scale bar: 1.5 s / 0.5 nA.
6. The activation I-V curve of voltage-gated potassium current in Go6893 WT and *Nlgn1* KO D2-MSNs.

G. Sample traces of Ca^2+^ signals of *Nlgn1* KO dorsal striatal D2-MSNs before and after Go6893 injection. Scale bar: 10 s / 1% df/f_0_.

H-I. Statistical graphs of the amplitude (H) and frequency (I) of Ca^2+^ events of *Nlgn1* KO dorsal striatal D2-MSNs before and after Go6893 injection.

Data represent mean ± SEM; Two-tailed unpaired t-test for panel B, repeated two-way ANOVA with Fisher's LSD post hoc test for panel D and F, two-tailed paired t-test for panel H and I. For panel B, dots represent individual neurons, for panel H-I, dots represent individual mice. *p < 0.05, **p < 0.01. Also see Table S2.
